# Extracellular Control of Radial Glia Proliferation and Scaffolding During Cortical Development and Pathology

**DOI:** 10.3389/fcell.2020.578341

**Published:** 2020-10-16

**Authors:** Julien Ferent, Donia Zaidi, Fiona Francis

**Affiliations:** ^1^Inserm, U 1270, Paris, France; ^2^Sorbonne University, UMR-S 1270, IFM, Paris, France; ^3^Institut du Fer á Moulin, Paris, France

**Keywords:** apical radial glia, cortical development, neuronal migration, scaffold, cell-cell interaction, cell signaling, extracellular matrix

## Abstract

During the development of the cortex, newly generated neurons migrate long-distances in the expanding tissue to reach their final positions. Pyramidal neurons are produced from dorsal progenitors, e.g., radial glia (RGs) in the ventricular zone, and then migrate along RG processes basally toward the cortex. These neurons are hence dependent upon RG extensions to support their migration from apical to basal regions. Several studies have investigated how intracellular determinants are required for RG polarity and subsequent formation and maintenance of their processes. Fewer studies have identified the influence of the extracellular environment on this architecture. This review will focus on extracellular factors which influence RG morphology and pyramidal neuronal migration during normal development and their perturbations in pathology. During cortical development, RGs are present in different strategic positions: apical RGs (aRGs) have their cell bodies located in the ventricular zone with an apical process contacting the ventricle, while they also have a basal process extending radially to reach the pial surface of the cortex. This particular conformation allows aRGs to be exposed to long range and short range signaling cues, whereas basal RGs (bRGs, also known as outer RGs, oRGs) have their cell bodies located throughout the cortical wall, limiting their access to ventricular factors. Long range signals impacting aRGs include secreted molecules present in the embryonic cerebrospinal fluid (e.g., Neuregulin, EGF, FGF, Wnt, BMP). Secreted molecules also contribute to the extracellular matrix (fibronectin, laminin, reelin). Classical short range factors include cell to cell signaling, adhesion molecules and mechano-transduction mechanisms (e.g., TAG1, Notch, cadherins, mechanical tension). Changes in one or several of these components influencing the RG extracellular environment can disrupt the development or maintenance of RG architecture on which neuronal migration relies, leading to a range of cortical malformations. First, we will detail the known long range signaling cues impacting RG. Then, we will review how short range cell contacts are also important to instruct the RG framework. Understanding how RG processes are structured by their environment to maintain and support radial migration is a critical part of the investigation of neurodevelopmental disorders.

## Introduction

The cerebral cortex is an intricate brain structure responsible for many precise functions such as thinking, decision making and long term memory, and is required for the final processing of sensory inputs and motor control. These functions rely on the way the neuronal network is precisely organized. The structure of the cortex is composed of different layers of neuronal subtypes (Taverna et al., 2014; De Juan Romero and Borrell, 2015). In the mouse, these layers are established during embryonic development in an inside-out manner via the successive migration of young neurons generated directly or indirectly from apical radial glial cells (aRGs) in the ventricular zone (VZ) to their final location in distinct superficial regions (Rakic, 1972; Kriegstein and Gotz, 2003). aRGs have a particular morphology as they grow processes that extend from the apical to the basal side of the cortex. In both rodent and primate, aRGs generate further basal intermediate neurogenic progenitors (IPs) residing in the subventricular zone (SVZ). In gyrencephalic species such as humans and other primates, neurons can also be generated from basal radial glia (bRGs), also called outer radial glia (oRG), which are distributed in an outer SVZ (Penisson et al., 2019; Matsumoto et al., 2020). bRGs can extend processes to the apical, the basal or both surfaces of the cortex (Betizeau et al., 2013). In all situations their structure provides a linear support for neuronal migration. Therefore, RGs are not only the source of neurons during embryonic cortical development but also the scaffold necessary for their proper distribution throughout the expanding cortex. The formation and maintenance of the RG scaffold is essential for the correct positioning of neurons and thus, the organization of the neuronal network.

**FIGURE 1:**
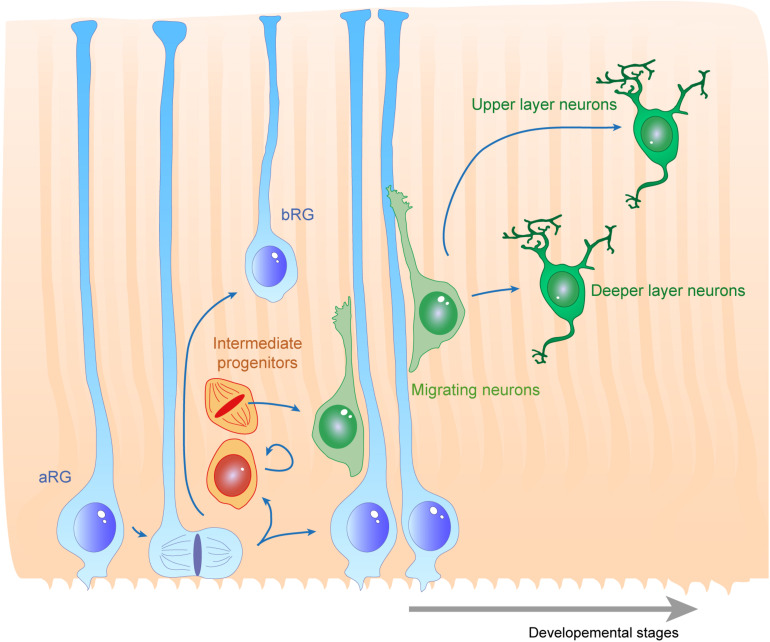
Radial glias function as both the source and the support of newborn neurons in the developing cortex. Apical radial glia (aRG) extend an apical process reaching the ventricular surface, where they expose their primary cilia, as well as a basal process reaching the cortical surface. Basal radial glia (bRG) have their cell bodies located in more basal areas of the cortical wall. Apical and basal processes from these cells (blue) establish the scaffold across the whole cortical wall. RGs undergo cell division, giving birth to a daughter cell which can be either another RG (apical or basal – symmetric division) or a basal progenitor (asymmetric division, intermediate progenitors are represented in orange). These cells give rise to migrating neuroblasts (green) which move along RG basal processes to reach their final position within the cortical layers. First deep layer neurons are generated, then upper layer neurons are born.

Several cellular processes are important to consider for proper RG morphology. As they are dividing and self-renewing progenitors, RGs have been widely studied in the context of the mechanisms underlying their proliferative features (Taverna et al., 2014; Uzquiano et al., 2018). This will have an impact on the density of fibers available for supporting migration. RGs (e.g., expressing factors such as Pax6, Sox2, Hes5) can self-renew via symmetric divisions but can also carry out asymmetric divisions giving rise to different progeny including Tbr2 + IPs (Figure 1; Taverna et al., 2014; De Juan Romero and Borrell, 2015). RGs are also able to directly produce neurons. In particular, cell intrinsic mechanisms acting on mitotic spindle orientation or nucleokinesis via cytoskeletal or polarity proteins are tightly linked to daughter cell production and fate. At the structural level, how the radial processes critical to RG function are created, modulated or maintained relies on additional molecular mechanisms, which is the topic of this review.

**FIGURE 2:**
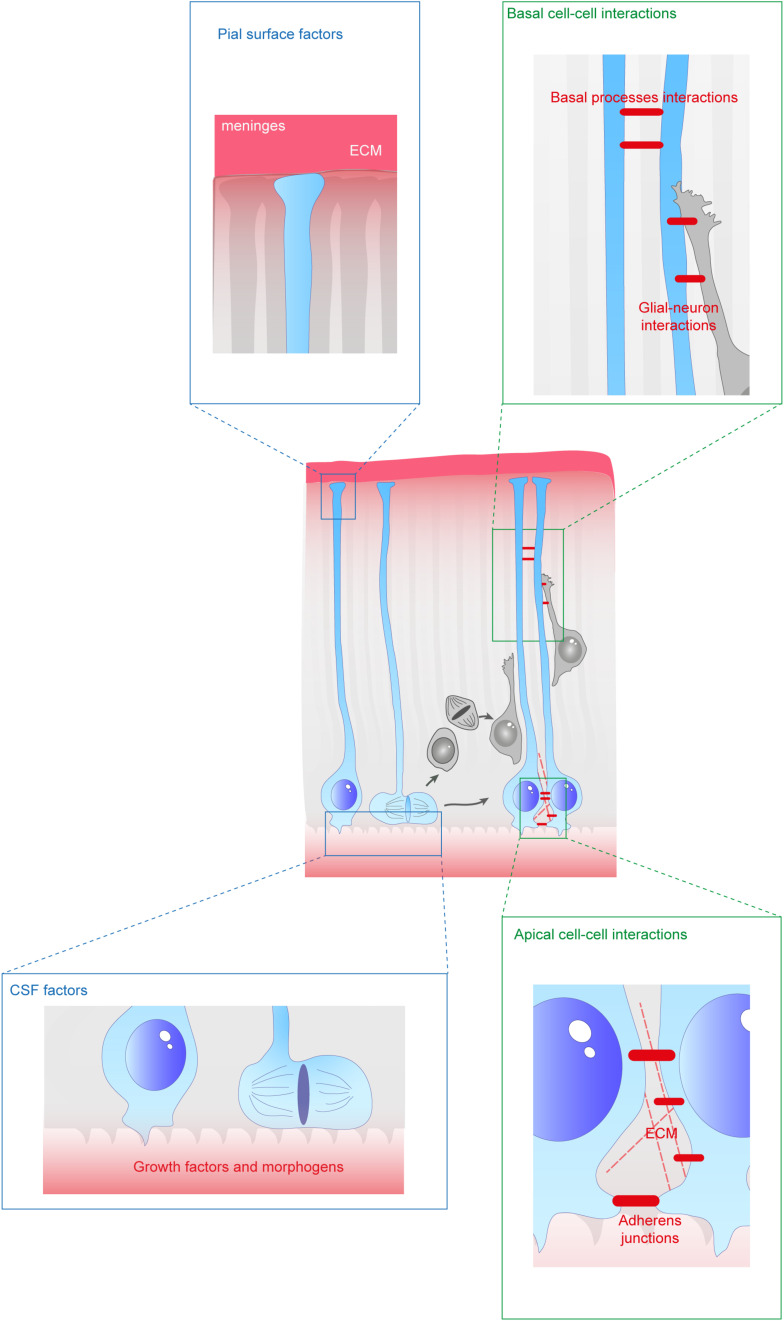
Extracellular factors controling the scaffolding of RGs. RGs are exposed to a variety of extracellular cues. These signals can be secreted molecules (blue boxes) or received directly from other cells (green boxes). In apical regions aRGs receive signals from the eCSF as their cell bodies and primary cilia are in contact with the ventricles. They also establish contacts between themselves and with the extracellular matrix (ECM). In basal regions, RG basal processes are exposed to secreted cues from the meninges and from already differentiated neurons. These interactions can occur while neurons are migrating along them. Basal processes also exhibit interactions between themselves.

Since aRGs are structured in a very defined way, with their soma and apical processes at the border of the ventricle, they are exposed to many secreted factors from the embryonic cerebrospinal fluid (eCSF). In particular, the primary cilium extends inside the ventricle and this is a crucial signaling center for the activation of numerous molecular cascades (Sarkisian and Guadiana, 2015). At the level of their cell bodies, aRGs and bRGs are both exposed to cell−cell and cell−environment interactions. They interact with each other as well as with additional cell types such as IPs or neurons. Importantly they interact with the surrounding extracellular matrix (ECM). For example, human bRGs have been shown to produce specific proteins which interact with the ECM in their basal position (Pollen et al., 2015). Finally, their basal processes are also exposed to external signals throughout the intermediate zone (IZ), cortical plate (CP), marginal zone (MZ), and at the pial surface (Figure 2). First, we will review the different secreted molecules involved in the establishment or maintenance of RG morphology from the eCSF or in the ECM. Then, we will describe how short range interactions between cells are essential for these processes. Finally, we will detail the impact of relevant molecular players on the origin and evolution of several human neurodevelopmental diseases.

## Role of Secreted Proteins Derived From the CSF in the Formation and Maintenance of the RG Scaffold

### Secreted Factors From the Embryonic Cerebrospinal Fluid (eCSF)

The cortex develops primarily from the neuroepithelium during embryonic development. Between E8.5 and E9.5 in mice, the neural tube closes, forming the ventricular cavity in which the amniotic fluid is sequestered and forms the basis for the eCSF (Lowery and Sive, 2009). Later during mouse brain development, the choroid plexus arises and secretes many factors, modifying the composition of the ventricular fluid (Chau et al., 2015). The deepest apical region of the developing brain is composed of neuroepithelial-derived aRG progenitors from E10.5. These aRGs are exposed to a variety of secreted factors from the ventricle during development. Proteomics analyses of the CSF indicates that the precise composition of secreted molecules varies during development. For instance, the concentration of Bone Morphogenic Proteins (BMPs) is higher in the amniotic fluid than in the eCSF, Sonic Hedgehog (Shh) concentration is higher in the eCSF at the beginning of aRG development (E10.5) and decreases thereafter, whereas the concentration of retinoic acid (RA) is higher at later stages (E14.5) (Chau et al., 2015). These variations in composition are required to induce the production of RGs (Sox2+) at the right time during the formation of the cortex (Chau et al., 2015). These data also suggest that certain secreted proteins or combinations of proteins in the eCSF during murine corticogenesis are required for evolving aspects of RG production and maintenance.

The composition of secreted factors in the eCSF not only changes with developmental stages but is also specific to different ventricles. Indeed, the different choroid plexus tissues present in each ventricle develop in a sequential manner (Lehtinen et al., 2011). Firstly, the choroid plexus from the fourth ventricle appears (E11 in the mouse), then the choroid plexus develops in the lateral ventricles (E12) and lastly, it develops in the third ventricle by E14. Each type of choroid plexus will express a different panel of secreted factors. For example, Shh is mainly produced in the fourth ventricle by the choroid plexus close to the hindbrain (Huang et al., 2010), whereas many other proteins are found only in the lateral ventricles (Zappaterra et al., 2007). More recently, proteomics data were integrated with RNA sequencing datasets, comparing telencephalic and hindbrain choroid plexuses (Lun et al., 2015). This spatial heterogeneity of their secretomes argue in favor of a precise and specific regulation of different brain areas. Overall, the eCSF plays multiple important roles in the formation of the nervous system (for review, Fame and Lehtinen, 2020). In this part of our review, we will describe the functional role of the main secreted factors present in the eCSF for the maintenance of RGs and therefore the formation of the RG scaffold.

### Growth Factors

**FIGURE 3:**
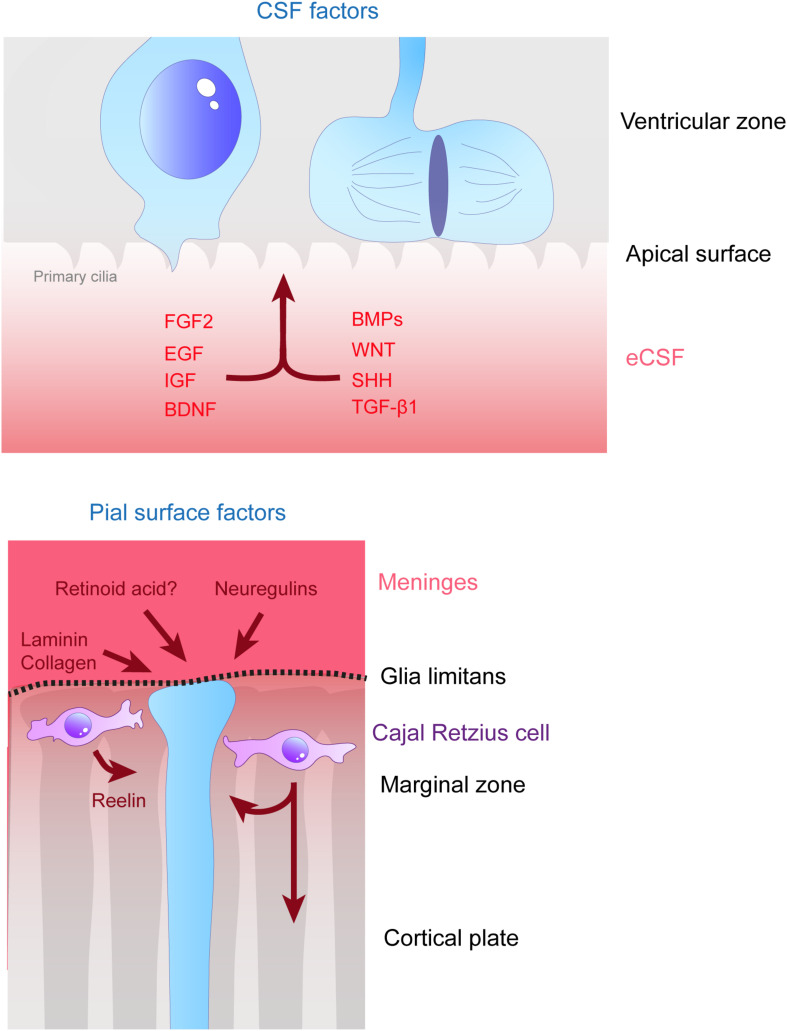
Remote extracellular factors controling the scaffolding of RGs. Some of the extracellular cues controlling RG development are produced and secreted from relatively remote locations. Here are represented the factors present in the CSF (upper schema) which are detailed in this review, namely FGF2, EGF, IGF, BDNF, BMPs, WNT, SHH, and TGF- β1. On the bottom schema, extracellular cues derived from the meninges and acting on the extremities of basal processes are depicted, namely, laminin, collagen, neuregulins and retinoid acid. Cajal Retzius cells (in purple) are migrating cells which in early stages of development tangentially move in the MZ of the developing cortex. These cells are a source of Reelin amongst other molecules which influence RG scaffolding.

**FIGURE 4:**
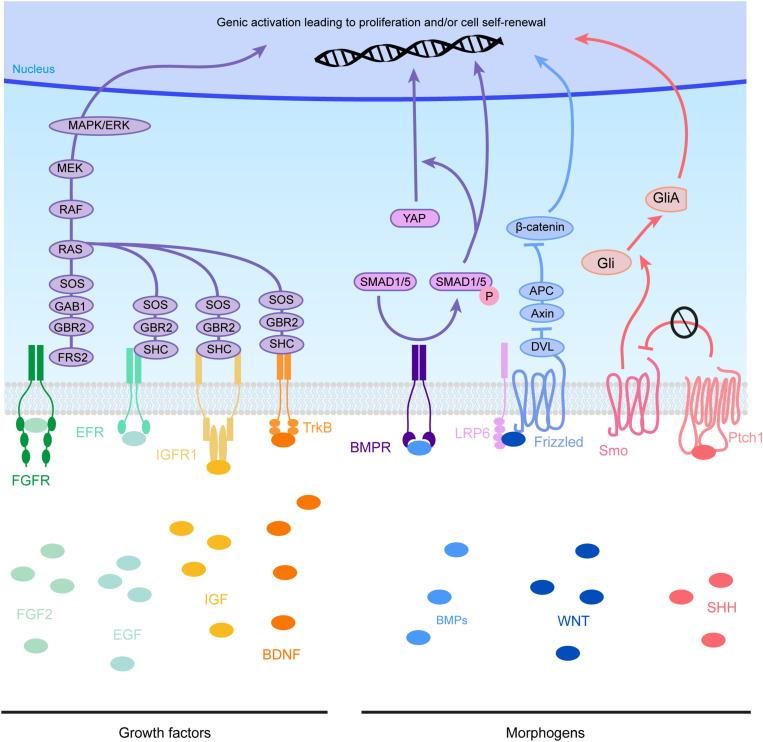
Molecular pathways triggered by eCSF-derived factors. The growth factors found in the eCSF are mainly known to trigger the mitogen-activated protein kinases (MAPK) pathway (also known as the RAS-RAF-MEK-ERK pathway). This molecular signaling pathway is involved in the regulation of several essential cellular processes such as proliferation, differentiation, survival and death. BMP receptors (BMPR) activate the phosphorylation of SMAD1/5, which can activate directly transcription of target genes or act via the translocation of YAP into the nucleus. WNT molecules activate the Frizzled receptors and LRP6 co-receptors which will allow Disheveled (DVL) to inhibit the Axin-APC complex. This complex is a major inhibitor of β-catenin. Therefore, upon WNT activation, β-catenin is free to be directed into the nucleus to activate its target genes. Finally SHH binds to its receptor Patched1 (Ptch1), which then releases the 7 transmembrane protein Smoothened (Smo) from its inhibition. Smo activation triggers the cleavage of Gli transcription factors into their active form (GliA). GliA is then enriched in the nucleus to allow transcription of target genes (such as Cyclin D1 or Gli itself).

As mentioned in the previous section, several cytokines are found in the eCSF. Different types of molecules can be found within this family such as growth factors like Transforming Growth Factors (TGF, developed later on in this review). But not all of these cytokines have a direct effect on radial scaffolding. For example, chemokines are best known for their action on neurons (Zhu and Murakami, 2012). On the other hand growth factors are diffusible cytokines widely known to activate RG proliferation and/or sustain cell survival. Therefore, we first provide a non-exhaustive list of eCSF-derived growth factors (Figures 3, 4 and Table 1) necessary for cortical development and in particular for the integrity of the RG scaffold.

**TABLE 1:** Non-exhaustive list of proteins influencing RG scaffolds during cortical development.

Protein	Localization	Implication in RG scaffold	Phenotype	References
**Afadin**	Apical endfeet	Apical process arrangement AJ maintenance	Apical process irregularly arranged Loss of AJ markers	Yamamoto et al., 2015
**aPKCλ (Atypical protein kinase C λ)**	Apical endfeet	RG polarity Apical process maintenance	Apical process retraction RG detachment	Fumiyasu et al., 2006
**APC (adenomatous poli C)**	RG tips Soma	Maintenance and extension of RG processes Scaffold polarity	Mis-oriented scaffold (basal process not directed at pial surface) Shorter processes	Yokota et al., 2009
**Arp2/3 (Actin Related Protein 2/3)**	Basal/apical endfeet Soma Nucleus	Formation and maintenance of AJs	Shorter RG processes and misoriented Lower speed of basal process formation Ventricular surface is altered	Wang P.S. et al., 2016
**Bone morphogenic proteins/SMADs**	eCSF Meninges Hem	Control of neurogenesis	Premature differentiation Thinner cortex/microcephaly	Najas et al., 2020
**Brain Derived Neurotrophic Factor**	eCSF RG Cajal-Retzius cells	RG self-renewal	Decrease of RG proliferation	Bartkowska et al., 2007
**Cdc42 (Cell division control protein 42)**	Leading process (basal fiber)	Basal process growth Inter−radial fiber interactions	Shorter basal process Decreased contacts between RG fibers	Yokota et al., 2010
**Ece2 (endothelin converting enzyme-2)**	RG apical compartment Cortical plate	Apical process maintenance RG morphology Ventricular surface integrity	Loss of apical process Ventricular surface alteration Loss of radial morphology	Buchsbaum et al., 2020
**ECM components and receptors**	Pial surface VZ SVZ	Apical process integrity Basal process integrity RG morphology		Milev et al., 1996; Li et al., 2008; Loulier et al., 2009; Sittaramane et al., 2009; Okamoto et al., 2013; Buchsbaum et al., 2020
**Epidermal growth factor**	eCSF	Maintenance of RG identity and self-renewal		Burrows et al., 1997; Lillien and Raphael, 2000; Sun et al., 2005
**Fibroblast growth factor**	eCSF	Production and maintenance of RG	Decrease in cortical size	Dono et al., 1998; Raballo et al., 2000
**FSTL1 (Follistatin like-1)**	Pial basement membrane	RG basal process orientation Basal endfeet branching	RG basal process not parallel Less endfeet branched	Liu et al., 2015
**Glial growth factor**	Neuronal secretion	Basal process elongation	Loss of endfeet formation and disrupted morphology	Anton et al., 1997
**GSK3 (Glycogen synthase kinase 3)**	Leading process (basal fiber)	Basal process growth and orientation Whole scaffold morphology	Shorter basal process Basal process mis-oriented Scaffold morphology altered	Yokota et al., 2010
**Insulin-like Growth factors**	eCSF	RG proliferation	Neurogenesis decrease	Lehtinen et al., 2011
**N-cadherin**	AJs	AJ maintenance Apical process maintenance	RG detachment Apical process retraction Premature differentiation	Rousso et al., 2012; Wong et al., 2012; Das and Storey, 2014
**Neuregulins**	RG	Maintenance of RG proliferation and radial morphology	Reduced number of RG	Schmid et al., 2003; Nakagawa et al., 2019
**Notch**	RG	RG identity Promotion of radial morphology Increase expression of adhesion proteins	Premature differentiation Overexpression: Radial morphology increased Adhesion protein expression increased	Li et al., 2008; Yoon et al., 2008
**Numb/Numbl**	Apical endfeet	Radial polarity Apical process maintenance AJ maintenance	Altered ventricular surface Loss of radial polarity Loss of apical process	Rasin et al., 2007
**Plekha7**	Apical endfeet	Apical process maintenance Apical contact integrity	Loss of apical contact Apical process retraction	Tavano et al., 2018
**Reelin**	Cajal-Retzius cells	Maintenance of RG morphology	RG process branching defects	Hartfuss et al., 2003; Schaefer et al., 2008; Chai et al., 2015
**Sonic Hedgehog**	eCSF and interneurons	Radial glia proliferation	Reduction in RG number	Komada et al., 2008; Dave et al., 2011; Wang L. et al., 2016
**TAG-1 (Transient axonal glycoprotein-1)**	Basal region	Basal process maintenance	Basal process loss Basal process retractation	Okamoto et al., 2013
**Transforming growth factor β 1**	eCSF	Control of RG morphology and processes	ND	Stipursky et al., 2014
**Wnt**	eCSF	RG self-renewal RG radial morphology	Basal process disruption Premature differentiation	Woodhead et al., 2006; Nakagawa et al., 2017

*The column phenotype takes into account results of experiments done when the protein is lacking (cKO, KO, pharmacological inhibition etc.) Proteins are presented in alphabetical order.*

Multiple **Fibroblast growth factor** (FGF) ligands are expressed in the developing telencephalon. At early stages (E10–E12), FGFs 8, 17, and 18 are expressed in the frontal midline area where they act as morphogens (see section “Morphogens” below). In the ventral telencephalon, FGF15 is expressed (Rash and Grove, 2006; Cholfin and Rubenstein, 2007; Hebert and Fishell, 2008), whereas in dorsal regions, FGFs 2, 9, and 10 are expressed (Vaccarino et al., 1999; Raballo et al., 2000; Sahara and O’Leary, 2009). Here, we focus on FGF2, which increases the total number of neurons in the mouse cerebral cortex and promotes self-renewal of cortical progenitor cells (Vaccarino et al., 1999; Raballo et al., 2000).

Fibroblast growth factor 2 is one of the most important growth factors for the production and maintenance of RGs during cortical development. Initially, FGF2 proteins were found present in the VZ of the murine developing cortex by immunohistochemistry (Dono et al., 1998). The source of the protein was not clearly defined but later the protein was detected in chicken eCSF (HH25) by western blot experiments (Martin and Groves, 2006), suggesting that it might be produced remotely and captured at the ventricular surface. In this study, authors show that the actual origin for FGF2 production in the chick embryo is the notochord, the mesonephros, the hepatic primordia and the brain neuroectoderm. The receptor for FGF2 (FGF2R) is expressed in the mouse VZ (E14.5) as shown by *in situ* hybridization (Dono et al., 1998). More recently single cell RNA-seq data shows that the *Fgf2r* gene is expressed in mouse RGs (Telley et al., 2019), suggesting that these cells can receive the FGF2 signal from the eCSF. The cortex of *Fgf*2 mutant mice is thinner and there is abnormal distribution of neurons in the cortical wall. Indeed, pulse chase analyses indicate an increase of neurons generated at E14.5 in the deep layers of the cortex (Dono et al., 1998). This suggests a defect in the ability of these cells to colonize their final target place in more superficial layers. Defects in proliferation were also identified in *Fgf2* KO embryonic cortices in a separate study explaining the decrease in the size of the cortex (Raballo et al., 2000). This is in agreement with the fact that FGF2 is one of the major factors necessary for the renewal of RGs *in vitro* (Sun et al., 2011). The knockout (KO) of *Fgfr* genes in the anterior neural plate using Foxg1-Cre, inhibits the formation of the telencephalon, leaving just the midline (Paek et al., 2009). When Fgfrs are removed only from RG, their development is impaired resulting in lower numbers of *Pax6* and *Hes5* + cells (Kang et al., 2009). These combined data show how crucial FGF2 is for the maintenance of RGs and therefore, the formation of the cortex. Moreover, gain of function experiments performed by *in utero* injection of FGF2 first in the telencephalic ventricles of rat embryos at E15 (Vaccarino et al., 1999), then in mouse embryos at E11.5 (Rash et al., 2013), induces an increase in proliferation. When FGF2 signaling is overactivated locally by these manipulations, this induces the formation of gyri in the mouse cortex (Rash et al., 2013). Although gyrification can be associated with the appearance of bRGs during evolution (Penisson et al., 2019), in this case FGF2 injections did not appear to increase the proportion of bRGs in the cortical wall. This suggests that FGF2 modified the development of the architecture of the cortex via other unknown mechanisms. At the molecular level, FGF2 triggers the mitogen-activated protein kinase (MAPK) pathway to induce cell cycle and proliferation (for review, Iwata and Hevner, 2009, Figure 4). FGF2, as well as Notch signaling, can also induce calcium (Ca2+) bursting which can support communication along the RG fiber (Rash et al., 2016). Indeed, both along the RG fiber and the communication with neurons can be mediated by calcium waves, in a bidirectional manner.

Fibroblast growth factor 2 can be used in culture in combination with **Epidermal growth factor** (EGF). Indeed, the action of EGF on cortical progenitors has been studied for many years (Burrows et al., 1997; Lillien and Raphael, 2000). Recently, FGF2 and EGF were shown to regulate self-renewal of rat cortical progenitors in organotypic cultures *in vitro* (Lamus et al., 2020). These two growth factors can activate the same molecular pathways to initiate proliferation. The action of FGF2 and EGF is not a simple synergy since FGF2 can modulate the responsiveness of RGs to EGF (Lillien and Raphael, 2000). RGs are first responsive to FGF2 alone and later during cortical development, start to be also responsive to EGF (Ciccolini and Svendsen, 2001). Moreover, the effect of EGF on the proliferation of cortical progenitors is dose-dependent (Nelson et al., 2008). The combined action of EGF and FGF2 is therefore essential for the development of RGs and their maintenance during the development of the cortex. At the beginning of mouse cortical expansion (e.g., E13), the EGF receptor (EGFR) is present in the VZ and SVZ (Sun et al., 2005). At the cellular level, EGFR has been found to be localized asymmetrically in dividing RGs, controlling the fate of daughter cells. The cell inheriting EGFR is the daughter cell retaining proliferative capacity and glial markers (Sun et al., 2005). This indicates that RGs need to keep their ability to respond to EGF in order to maintain their progenitor identity. EGF signaling is therefore required for the maintenance of the RG scaffold during cortical development. Certain studies have investigated the mechanisms controlling the expression of EGFR. First the ganglioside GD3 was identified as an EGFR partner, responsible for its sustained expression in cortical progenitors *in vitro* (Wang and Yu, 2013). More recently, the expression of miR-129-5p, modulated by choline availability in the microenvironment, was shown to inhibit the expression of EGFR, thus impacting RG maintenance and cortical development (Trujillo-Gonzalez et al., 2019). All these data underline the essential role for EGF in the maintenance of RGs necessary for cortical development.

The **Insulin-like Growth factors** (IGF1 and IGF2) are a group of hormones which are present in the eCSF (Salehi et al., 2009; Zappaterra and Lehtinen, 2012; Bueno et al., 2020). IGF2 concentration in rat eCSF increases from E16 to E19 (Lehtinen et al., 2011). Gain of function experiments such as IGF1 overexpression in mouse embryo showed that this hormone induces a shortening of the cell cycle, acting in particular on S-phase. This increase in the speed of proliferation is linked to cortical hyperplasia, which is an increase in global cortical size via an increase in cell number (Hodge et al., 2004; Popken et al., 2004; Mairet-Coello et al., 2009). Both *in vivo* and *in vitro* data show that IGF2 can induce cortical growth by stimulating RG proliferation (Lehtinen et al., 2011). The reverse result is observed in *Igf2* KO mice which present a neurogenesis decrease affecting the production of neurons destined for the upper cortical layers. At the apical membrane level, CSF-derived IGF2 binds to primary cilia of RGs, where IGF receptors are localized. IGF1 receptor (IGF1R) is the main receptor allowing IGFs to trigger proliferation (Zappaterra and Lehtinen, 2012). Like FGF2 and EGF, IGF can trigger the MAPK pathway but can also activate a non-canonical pathway via Gβγ signaling, which regulates the timing of the cell cycle (Yeh et al., 2013). All of these data explain why the *Igf1r* conditional knockout (cKO) in neural precursors leads to microcephaly (Lehtinen et al., 2011).

**Brain Derived Neurotrophic Factor** (BDNF) has the particularity of being expressed directly by RGs and also by Cajal-Retzius cells (Fukumitsu et al., 1998). The role of BDNF on RGs has been investigated by both injection of BDNF itself directly in ventricles at mouse E13.5 (Fukumitsu et al., 2006) and also by overexpression of *Bdnf* in cortical precursors *in vivo* via *in utero* electroporation (Bartkowska et al., 2007). Both strategies led to an increase in proliferation. BDNF is one of the ligands which can activate the tropomyosin receptor kinase B (TrkB) receptor. The loss of function of its gene (*Ntrk2*) in the mouse was achieved by different approaches such as by short hairpin RNAs (shRNAs) or by expression of a dominant-negative variant of TrkB. Blockade of TrkB signaling elicited a decrease in RG self-renewal (Bartkowska et al., 2007). TrkB receptors can be phosphorylated, which will activate the MAPK or phosphoinositide 3-kinase (PI3K) pathways (Numakawa et al., 2018). Both pathways are implicated in different functions of RG behavior during cortical development. PI3K is important for RG survival whereas MAPK activation is required for the production of neurons (Barnabe-Heider and Miller, 2003). Therefore, the pathway linking BDNF, TrkB and PI3K is essential for the maintenance of the RG scaffold, indeed activation of the BDNF-TrkB-MAPK axis can lead to premature RG differentiation into neurons via the activation of BMP7 (Ortega and Alcantara, 2010 and see below). BDNF can also activate Anoctamin 1 (ANO1), a Ca2+-activated chloride channel which is expressed in RGs (Hong et al., 2019). The growth of RG basal processes is dependent on the activity of this channel as its loss of function disrupts the extension of RG protrusions. ANO1 overexpression inversely increases this process (Hong et al., 2019). The lack of basal process growth in *Ano*1-deficient mice leads to disorganized cortical layers and microcephaly (Hong et al., 2019).

**Transforming growth factor β 1** (TGF-β1) is a cytokine which is involved at many levels of neuronal development (Meyers and Kessler, 2017). TGF-β1 is present in the VZ of the developing cortex (Mecha et al., 2008) and its receptor, TGFRII, is highly expressed by RGs (Stipursky et al., 2014). Although its role is mainly associated with the differentiation of RGs into either neurons or glia (Stipursky et al., 2012, 2014), injection of TGF-β1 directly into the embryonic ventricles at E14 induces drastic changes in RG scaffold morphology. Basal processes seem shorter and disorganized. In fact, TGF-β1 triggers early transition of RGs into astrocytes which alters their morphology from radial to multipolar. Interestingly, these effects are similar to the action of a morphogen, as described in the next paragraph, more than a growth factor.

Thus, different growth factors play apparently critical roles influencing the formation and maintenance of RG scaffolds.

### Morphogens

Contrary to growth factors which are known classically to act at the proliferation level, morphogens are instead also associated with an action at the differentiation level and to control cell fate decisions (Briscoe and Small, 2015). We review here the known roles of morphogens in the maintenance of RGs and therefore the RG scaffold (Figures 3, 4 and Table 1).

Certain members of the TGF family, e.g., the **bone morphogenic proteins** (BMP), have important roles in the maintenance of RG scaffolding. *In vitro* experiments on cultures of RGs indicated that BMP signaling is involved in the control of neurogenesis (Li et al., 1998; Mabie et al., 1999). Bmp7 has been detected in the meninges, hem and also in the eCSF (Segklia et al., 2012). When Bmp7 is removed from the mouse brain, this leads to reduced cortical thickness and number of neurons at E14.5. On the other hand, when Bmp signaling is activated by expressing a constitutively active form of its receptors (Bmpr1a or Bmpr1b), over proliferation and defects in global morphology are observed in the developing cortex (Panchision et al., 2001). In particular, folds can be seen at the brain surface, suggesting differences in RG scaffolding. More recently, the implication of Smad1/5 (canonical BMP transcription factors) was revealed by loss of function experiments in both mouse and chick (Najas et al., 2020). In these models, RG maintenance was disrupted, and premature differentiation occurred, which leads to a microcephaly phenotype. The consequences of KO were assessed on neurogenesis but not the RG scaffold *per se*. However, SMADs are likely to regulate neurogenesis by modulating YAP (Yes-associated protein) activity (Najas et al., 2020), since decreasing SMAD1/5 leads to a decrease in YAP translocation into RG nuclei. This is crucial for cortical development as Hippo signaling has been linked to apical RG surface integrity and adhesion (Roy et al., 2019). Moreover, KO of YAP and TAZ, a transcriptional coactivator with PDZ-binding motif, can rescue genetically driven (via a *Pard3* deletion in the mouse) cortical heterotopia associated with detached RGs and higher YAP levels (Liu et al., 2018, see also section “Further Factors Identified via Human Pathology” *Human pathology*). Overall, these data indicate an important role of BMPs via their activation of SMADs in the control of RG behavior during cortical development.

The **Wnt** morphogen is implicated at many levels of neural system development and in particular in the cortex (Harrison-Uy and Pleasure, 2012). Wnt proteins are present and active in the eCSF where they are transported by lipoprotein particles (Johansson et al., 2013; Kaiser et al., 2019). Many different studies point to the role of Wnt as an essential factor in maintaining RG identity and self-renewal. A scaffolding disruption phenotype as well as proliferative defects are described in the developing hippocampus in the *Lrp6* gene mouse KO, one of the most important Wnt co-receptors (Pinson et al., 2000; Zhou et al., 2004; Wang Y. et al., 2016). Concerning the intracellular signaling triggered by Wnt, the canonical pathway relying on β-catenin inhibits neurogenesis by keeping RG undifferentiated (Woodhead et al., 2006; Wrobel et al., 2007; Mutch et al., 2010; Munji et al., 2011). β-catenin can be involved in different cellular processes such as cell-cell adhesion in addition to its transcriptional role. In one study, the authors specifically abrogated β-catenin’s transcriptional role by expression of a truncated form of this molecule in the telencephalon (Draganova et al., 2015). This study showed that Wnt/β-catenin signaling regulates a network of transcription factors involved in specific stages of cortical development including Dach1, Eya2, Etv5, and also Nfix (Draganova et al., 2015). In the Wnt/β-catenin pathway, Adenomatous polyposis coli (APC) is a regulator of β-catenin driving its degradation in the absence of Wnt binding at the membrane (Nelson et al., 2015). In an APC conditional KO in mouse RGs, the scaffold of basal processes is disturbed (Nakagawa et al., 2017). It is also interesting to note that Wnt signaling has been implicated in the maintenance of basal progenitors via the regulation of N-myc (Kuwahara et al., 2010). Therefore, since several Wnt molecules are expressed at different levels of the developing cortex (i.e., Wnt7a at the apical surface and Wnt7b in the basal parenchyma), it is possible that this morphogen can regulate the RG scaffold throughout the cortex and even in superficial regions.

The presence of the **Sonic Hedgehog** morphogen (Shh) ligand in the developing cortex has been known for several years (Komada et al., 2008). Shh is a well-known morphogen which can control a lot of different aspects of neurodevelopment at different locations of the nervous system (for review see Ferent and Traiffort, 2015). Shh is present in the eCSF, providing a source for the VZ, as identified by the ELISA method (Huang et al., 2010; Chau et al., 2015; Lun et al., 2015). Shh production occurs in cells of the choroid plexus of the fourth ventricle of the hindbrain (Huang et al., 2010) but not from the choroid plexus from the telencephalon (Lun et al., 2015). This would suggest that ventricular derived-Shh derived from the hChP would have to travel long distances to reach the ventricular wall of the developing cortex. Very recently, Shh secretion in the eCSF was linked to the ESCRT-III system (Endosomal sorting complex required for transport). Indeed, the *Chmp1a* (a gene coding for the charged multivesicular body protein *1a*, a subunit of the ESCRT complex) null mice present a decrease in the amount of Shh in the eCSF, correlated with a reduction in RG proliferation and the development of microcephaly (Coulter et al., 2018). This phenotype can be rescued when Shh signaling is genetically activated, showing that the ESCRT system is indeed upstream of Shh secretion. Migrating interneurons and Cajal-Retzius cells also produce Shh locally within the cortex (Dahmane et al., 2001; Flandin et al., 2011).

Several studies focused on the role of receptors or downstream signaling components of the Shh pathway during cortical development. Loss of function of the Smoothened Shh signaling activator in RG using GFAP-Cre or Nestin-Cre mice showed a decrease in proliferation, whereas activating the pathway via Patched1 receptor KO showed an increase (Dave et al., 2011; Wang L. et al., 2016). Overexpression of a constitutive form of Smo (SmoM2) increases the proportion of bRG in the developing cortex, suggesting a potential role for Shh signaling in the formation of bRGs (Wang L. et al., 2016). The role of the Patched1 co-receptor *Cdon* in cortical development has been highlighted by a loss-of-function study showing that deletion of Cdon leads to cortical microcephaly and reduction in RG proliferation (Zhang et al., 2006). At the molecular level, Shh controls the activity of Gli transcription factors to favor Gli2 activating forms over Gli3 repressor forms (Figure 4). Therefore, *Gli2* mutant mice present a decrease in RG proliferation (Palma et al., 2004) whereas Gli3 repressor form invalidation leads to an increase in cell cycle speed (Wilson et al., 2012). Suppressor of Fused (Sufu) is an important inhibitor of Shh signaling activity. Some ectopic progenitor clusters are detected in the cortical wall, showing over proliferation, when Sufu is conditionally knocked-out in the murine cortex (using Emx1-Cre) (Yabut et al., 2015). Ultimately, this leads to major defects such as a thinner cortex and strong differentiation disruption. Very recently, Yabut et al. showed that Sufu regulation of the Shh pathway controls the expression of *Fgf15* which is responsible for lineage progression of RGs (Yabut et al., 2020). This is a good example of how different extracellular cues can influence one another to modulate RG behavior.

Thus morphogens can have multiple effects but they are notable in their impact on RG structure, maintenance sand behavior.

## Secreted Factors From Close Range Cells

The eCSF is not the only source of secreted factors controlling the RG scaffold. Extracellular cues can be also sent from neighboring cells throughout the tissue. For example, the formation and maintenance of the basal process is dynamic (Yokota et al., 2010), with important information received from the meninges (Radakovits et al., 2009; Siegenthaler et al., 2009). This basal communication is not well known, including the mechanisms by which the meninges provide information to basal processes for their maintenance. Here, we provide examples of proteins involved in RG scaffold maintenance in response to extracellular cues produced locally within the developing cortex or from the meninges (Figure 3).

**Neuregulins** (NRG) play a major role in neuronal migration and RG integrity (Anton et al., 1997; Lopez-Bendito et al., 2006). In particular, mouse KO of *Nrg-1* leads to reduced cell numbers in primary cultures of embryonic progenitors (Schmid et al., 2003). NRG activates the v-Erb-a erythroblastic leukemia viral oncogene homolog (ErbB) family of tyrosine kinase receptors. ErbB2, 3, and 4 are expressed by RGs and are present along their basal processes (Schmid et al., 2003). Importantly, ErbB2 expression is specific to RGs and its loss of function in the mouse unbalances the astrocyte/RG population ratio by reducing the number of elongated RGs in the developing cortex (Schmid et al., 2003). ErbB2 interacts specifically with a redox active protein, Memo1 (Newkirk et al., 2018). Although Memo1 has been known for some time to be important for cell migration (Marone et al., 2004), its role in the branching and the maintenance of the RG scaffold was identified relatively recently (Nakagawa et al., 2019). A link has also been established between Nrg signaling and mGluR5 receptors. Indeed mGluR5 is coupled to the non-selective cation channel, canonical transient receptor potential 3 (Trpc3) (Louhivuori et al., 2015) and its loss of function in the mouse disrupts the formation of RG processes. This RG growth defect mediated by the mGluR5/Trpc3 signaling blockade can be rescued by Nrg/ErbB4 signaling showing that Nrg/ErbB4 is downstream of mGluR5/Trpc3 (Louhivuori et al., 2018).

**Retinoic acid (RA)** is a very well-known neurogenesis modulator. The particularity of this factor is that it is produced by different sources which could each impact cortical development. Although RA is secreted in the eCSF as described in chick (Alonso et al., 2011) and in zebrafish (Chang et al., 2016), its role on RG behavior has mainly been attributed to the meninges source (Siegenthaler et al., 2009). Indeed, when meninges are disrupted, limiting the supply of RA, or when a hypomorphic allele for the RA synthesizing enzyme Rdh10 is generated in the mouse, production of IPs is decreased (Siegenthaler et al., 2009). Nevertheless, this phenotype was not observed in *Rdh10* -/- mouse embryos (Chatzi et al., 2013; Haushalter et al., 2017), nor in conditional KO embryos for the other enzyme responsible for RA synthesis, Raldh2 (Haushalter et al., 2017). Therefore, it seems that although meninges-derived RA is important, its role with respect to RGs stills needs clarifying. The role of eCSF RA has also not yet been clearly identified.

Cajal-Retzius cells, present in basal regions in the MZ of the developing cortex, secrete, amongst other factors, **Reelin**, a glycoprotein which interacts extracellularly with receptors on migrating neurons (Sekine et al., 2014, see also section “Further Factors Identified via Human Pathology” *Human pathology*). When RG basal processes reach the MZ, they branch, however this branching is impaired in the *reeler* mutant mouse (deficient for Reelin, Chai et al., 2015). This indicates that besides its classical role influencing migrating neurons, Reelin may also control some aspects of RG morphology and influence the scaffolding (see also Hartfuss et al., 2003; Schaefer et al., 2008). Also, Reelin was linked to maintaining hippocampal RG integrity, since *reeler* tissue also showed precocious conversion of RGs to astrocytes, rescued by exogenous sources of Reelin (Zhao et al., 2004). Amongst the signals secreted by the meninges, **CXCL12** (chemokine (C-X-C motif) ligand 12) also called SDF1 (stromal cell-derived factor 1) can directly act on Cajal-Retzius cells and therefore indirectly modify the formation of RG scaffolding (Borrell and Marin, 2006). Briefly, CXCL12 controls tangential migration of Cajal-Retzius cells and disruption of its receptor CXCR4 leads to their displacement in deeper layers of the cortex, resulting in a dysplastic cortex (Paredes et al., 2006). Similarly in the hippocampus, CXCR4 invalidation also leads to severe phenotypes, including dentate gyrus granule neuron migration defects but also reduced proliferation of RG-like progenitors (Lu et al., 2002; Berger et al., 2007).

Bidirectional interactions between migrating neurons and RGs are essential for RG fiber growth. The **glial growth factor** (GGF), a soluble form of neuregulin, is expressed by migrating neurons along RG fibers and influences positively the growth of the RG fiber. In a pioneering study, Anton et al. (1997) provided evidence suggesting that the effect of GGF signaling on fiber elongation via ErbB2 is mediated through BLBP (brain lipid binding protein), an RG-expressed molecule (see also Hartfuss et al., 2003; Poluch and Juliano, 2010). It is thought that the rate of migratory neurons influences the lengthening of RG fiber, which also influences the rate of migratory neurons (Anton et al., 1997).

In the pial basement membrane (BM), a novel role for the secreted glycoprotein, **Follistatin like-1** (FSTL1) was identified in RG scaffolding (Liu et al., 2015). Indeed, authors showed that in embryonic mouse cortices, RG basal processes were not parallel and their endfeet less branched. Thus, they provide data suggesting that this protein is important for the basal but not the apical process and plays its role through a unique mechanism that does not include Cdc42 and GSk3β (Liu et al., 2015). This emphasizes the fact that multiple mechanisms are involved in the formation of the RG scaffold.

## Role of Cell to Cell and Cell to ECM Contacts in the Formation and Maintenance of the RG Scaffold and Proliferation

Because RGs extend across the whole cortical wall, they make numerous and various contacts. This section will focus on the impact of different contacts on their scaffold and their proliferative capacity.

### Adherens Junctions (AJs)

**FIGURE 5:**
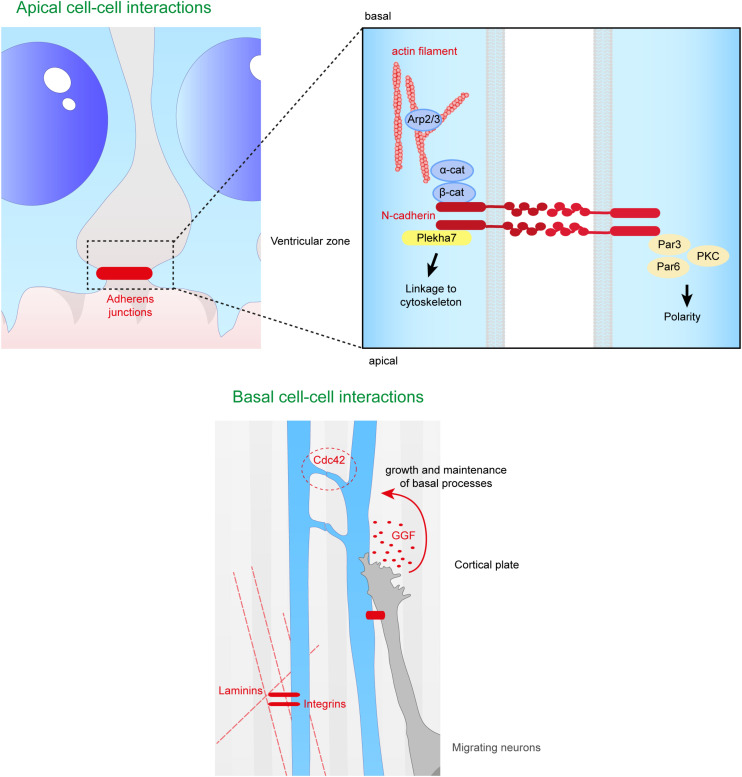
Close range contacts controling the scaffolding of RGs. RGs directly receive signals from neighboring cells such as other RGs or migrating neurons. On the top panel are depicted the cell–cell interactions occurring at the apical side of aRGs. Adherens junctions between aRGs are crucial for the maintenance of the scaffold. In the enlarged box is represented the binding of N-cadherins which can link extracellular contacts with the cytoskeleton (via Plekha7 or β-catenin) or with polarity proteins such as Par3, Par6, and PKC. On the bottom panel is illustrated basal cell–cell interactions. Basal processes of RGs can interact with each other inducing a Cdc42 response intracellularly. Neurons can also directly act on the glial scaffold by secreting factors such as GGF which controls growth and maintenance of basal processes. Finally, basal processes receive information from the extracellular matrix, especially via the interaction between intergins and laminins.

At the onset of neurogenesis, neuroepithelial cells become RGs and lose tight junctions, but AJs are maintained (Aaku-Saraste et al., 1996). These are composed of junctional complexes including N-cadherin, β catenin, α catenin and the cytoskeleton, which connect the apical regions of RGs to each other at the apical ventricular surface facing the eCSF (Figure 5).

The extracellular domain of **N-cadherin** enables the anchoring of the cells to each other, while the intracellular domain is connected to β and α catenins to link the AJ to the cytoskeleton. Therefore, this complex links the actin cytoskeleton to the plasma membrane to form cadherin mediated cell-cell adhesion sites (Drees et al., 2005; Nelson, 2008; Pokutta et al., 2008; Benjamin et al., 2010; Maiden and Hardin, 2011). Many studies (Miyamoto et al., 2015; Veeraval et al., 2020) emphasize the fact that N-cadherin based AJs are key elements for the development of cortical architecture. Several proteins involved in the maintenance of AJs, including afadin, as well as N-cadherin, αE catenin, β catenin, are essential for the formation and maintenance of the RG scaffold (Table 1).

First, RG N-cadherin based AJs are Numb-dependent (Kadowaki et al., 2007; Rasin et al., 2007). Numb acts as an inhibitor of Notch signaling and is localized in apical endfeet of RGs. Numb/Numbl (homolog of Numb) interact with cadherin-catenin complexes during cortical development and are essential to maintain N-cadherin AJs. When Numb and Numbl are lacking in mouse cortices, the ventricular surface is altered, and RGs lose their radial polarity and their apical process suggesting an essential role of Numb/Numbl in apical process maintenance (Rasin et al., 2007). Moreover, N-cadherin has been shown to be required to prevent RG delamination, apical process retraction and premature differentiation (Rousso et al., 2012; Das and Storey, 2014; Wang P.S. et al., 2016). The maintenance of cadherin based AJs enables the activation of the β catenin phosphodegradation complex (Gsk3β, APC, Axin) and so reduces its level in the cytoplasm (Maher et al., 2009). As mentioned in section “Secreted Factors From Close Range Cells,” β catenin is an effector of Wnt signaling, and is stabilized in the cell, influenced by the N-cadherin and Akt pathways. Interestingly, the presence of N-cadherin also allows Akt activity, and the phosphorylation of β catenin by Akt increases its translocation to the nucleus (Zhang et al., 2010; Zhang et al., 2013). These data further suggest a role of N-cadherin in the regulation of β catenin level and distribution. N-cadherin function is also impacted by the conditional KO of afadin in the cortex, and mice exhibit a double cortex (a normotopic cortex as well as a heterotopic cortex) due to detached RGs. In these mutants Yamamoto et al. (2015) showed that major proteins (including N-cadherin) of RG AJs are not maintained at E14.5, suggesting that afadin plays an essential role in the maintenance of the AJs in the apical processes. In this case, ectopic detached Sox2 + progenitors result. RG basal processes were not altered but the apical processes were irregularly arranged in the deficient embryonic mouse cortices. Interestingly, no defects in RG proliferation and differentiation were found in these mutants (Yamamoto et al., 2015), further suggesting a crucial role of basal attachment on RG proliferation (Uzquiano et al., 2018).

It is important to mention the **Arp2/3 complex** that is involved in the formation and maintenance of AJs. When Arp2/3 is conditionally deleted in the mouse, RG processes are shorter and mis-oriented (Wang P.S. et al., 2016). More precisely, the ventricular surface is altered with the presence of ectopic progenitors and the speed of formation of the basal process is also reduced. The Arp2/3 complex is an effector of β catenin, and establishes a link between the formation of the RG scaffold and the cytoskeleton (Wang P.S. et al., 2016). Cdc42 and RhoA are known to be upstream regulators of the Arp2/3 complex, and control both basal process extension and apical process adhesion (Cappello et al., 2006; Yokota et al., 2010).

Also, **Atypical protein kinase C λ** (aPKC λ) is a protein kinase present at the level of AJs during mammalian corticogenesis, and this protein forms complexes with polarity proteins Par6 and Par3. When aPKC is conditionally deleted in mouse cortex, apical processes of RGs retract more often and RGs detach from the ventricular surface. aPKC is indispensable for neuroepithelial cells to form AJs and maintain cell polarity in the neuroepithelium (Imai et al., 2006). Also, classical polarity proteins (Par3, Llg1) are phosphorylated by aPKC during RG polarity establishment (leading to the specific bipolar RG morphology) (Yamanaka et al., 2003) emphasizing a role of classical polarity proteins in this scaffolding. As mentioned above, *Pard3* conditional KO leads to detached RGs (Liu et al., 2018).

**Plekha7** is another protein associated with apical AJs, and is involved in the maintenance of the RG scaffold, preventing RG delamination. Tavano et al. (2018) showed the importance of this AJ-associated protein by forcing the expression of *Insulinoma-associated* 1 (*Insm*1) in RGs. Insm1 is a transcription factor that represses *Plekha*7 transcription. By forced expression of Insm1, there was an increased proportion of bIPs (multipolar cells) and bRGs, suggesting an alteration of the RG scaffold across the brain. Thus, when the level of Plekha7 is reduced by Insm1 forced expression, RGs lose their apical contact and their apical processe retracts (Tavano et al., 2018). This study confirms that AJ components are crucial for the RG scaffold and more specifically for apical process integrity.

Thus, perturbing or changing AJs influences RG attachment and this is one method by which apically detached RGs can arise (Penisson et al., 2019; Kalebic and Huttner, 2020). The fate of the detached cell can be variable (e.g., aberrant RG, bRG, IP or neuron) depending often on mutant conditions. Also, as described later in this review (see section “Further Factors Identified via Human Pathology” *Human pathology*), certain human cortical malformation gene mutations have been identified, related to further apical adhesion complexes. These data also emphasize the importance of RG apical contacts for the intact RG scaffold and correct neuronal migration.

### Extracellular Matrix (ECM) Components

The ECM is essential for corticogenesis and neural development (for review see Long and Huttner, 2019). In the embryonic cortex the ECM, composed of various proteins such as laminins, proteoglycans, dystroglycans and collagens, surrounds the cells (including RGs). Transcriptome analyses of human and mouse germinal zones in developing cortex (identified from laser microdissected material) showed that the variation of expression of ECM protein interactions and cell adhesion is likely to regulate the ability of neuronal progenitors to proliferate. Also, the expression profile of ECM proteins emphasizes differences between mouse SVZ (containing IPs and few bRGs) and human SVZ (oSVZ, iSVZ containing numerous bRGs and IPs) (Arai et al., 2011; Fietz et al., 2012). As already mentioned, further transcriptome analyses showed that an increase in the production of bRGs which no longer have contact with the eCSF, seems likely to require a modified ECM compared to ventricular aRGs (Pollen et al., 2015; Kalebic and Huttner, 2020). In a gyrencephalic model (ferret), it has been shown that inhibiting integrin (a major ECM receptor, Yamada and Sekiguchi, 2015) in the developing neocortex leads to a reduction in the number of bRGs (Fietz et al., 2010). Conversely, in the mouse increased expression of integrin increases the proliferation of basal progenitors (Stenzel et al., 2014). Recently, Kalebic et al. (2019) showed that the increased ability of bRGs to proliferate was associated with an increased number of RG processes. Indeed, increased processes allows the bRGs to multiply the reception of proliferative signals *via* integrin.

Radial glia basal processes extend across the cortical wall from the VZ to reach the pial surface (Rakic, 1972). These basal fibers are attached at the pial surface in part by **integrin−laminin-interactions**, allowing the migration of newborn neurons to reach their correct place in the CP (Graus-Porta et al., 2001; Belvindrah et al., 2007). Integrin−laminin interactions help anchor RG basal processes to the pial BM. Interestingly, laminin induces intracellular signaling via several receptors (e.g., as well as integrin, also dystroglycan, see section “Further Factors Identified via Human Pathology” *Human pathology*).

Related to this, **TAG-1**, for transient axonal glycoprotein-1, is a cell surface molecule expressed in the basal region of the cortical wall during embryonic development. This molecule also known as contactin-2 is essential for the maintenance of RG basal processes. Indeed, knockdown of TAG-1 in the mouse leads to basal process retraction and ectopic progenitors (Okamoto et al., 2013). The role of TAG-1 in basal RG process maintenance is cell autonomous, and knockdown does not affect apical surface integrity even if it increases mechanical stress in the ventricular zone. Indeed, the role of TAG-1 in the apical process is not well established. The mechanisms underlying its role in the basal RG fiber are not well defined but one hypothesis is that it is through the interaction of TAG-1 and basal lamina components such as Anoxin-1 and Laminin (Milev et al., 1996; Sittaramane et al., 2009; Okamoto et al., 2013).

Furthermore, *via* isolated stabilized RG clones, Li et al. (2008) showed that activated **Notch** promotes radial morphology, increases expression of BLBP (mentioned at the end of the section “Role of Secreted Proteins Derived From the CSF in the Formation and Maintenance of the RG Scaffold” as an actor in RG process elongation) and promotes RG adhesion on a laminin/nidogen complex. In this *in vitro* model, the authors observed increased expression of other adhesion proteins such as proteoglycans contributing to the brain ECM. Regarding these findings we can hypothesize that Notch action on radial morphology is likely to involve these ECM elements (Li et al., 2008). Also indirectly, Notch has been shown to be important in RG scaffolding. Indeed, the first step of the establishment of the RG scaffold is the maintenance of RG identity itself. Yoon et al. described data involving Notch signaling in the maintenance of the RG pool. By a specific deletion of *mind bomb 1* in mouse embryonic neuronal progenitors, Notch activation was inhibited and premature differentiation of RGs to IPs and neurons was observed (Yoon et al., 2008).

Loulier et al. (2009) demonstrated that the integrin-laminin interaction may act also in apical processes at the ventricular surface. Using blocking antibodies delivered into the cerebral ventricle *in utero*, preventing the fixation of laminin to its ligand β1 integrin, they observed detachment of RG apical processes, suggesting an apical role of the integrin-laminin interaction (Loulier et al., 2009). These findings provided evidence of the ECM’s role in RG bipolar shape and proliferative ability. More recent transcriptome and proteome analyses continue to contribute information concerning the ECM and different progenitor types including RGs (Fietz et al., 2012; Pollen et al., 2015; Buchsbaum et al., 2020; Kalebic and Huttner, 2020). For example, recently, interested in periventricular heterotopia (see section “Further Factors Identified via Human Pathology” *Human pathology*), Buchsbaum et al. (2020) provide novel information concerning the endothelin converting enzyme-2 (ECE2) gene and RG morphology. In cerebral organoids and the developing mouse cortex, they show that knockdown of *ECE2*/*Ece2* changes aRG morphology since these cells are less radial and bipolar. The ventricular surface was also altered, and aRG lose their apical process suggesting a role of this protein in apical RG processes. Interestingly, this ECE2-deficient phenotype is associated with ECM protein and receptor dowregulation.

Extracellular matrix components thus clearly have crucial roles in RG morphology and proliferative ability. This is a current exciting area of research which will further clarify the precise mechanisms involved.

### Interactions via Basal Processes (RG−RG, RG-Neuron)

As RGs are a physical support for post-mitotic neuron migration, it is clear that neurons and RG interact. This interaction provides to neurons a mechanical support for migration but also a way to communicate with RGs that can influence the migration process. It is thus not only the integrity of the RG scaffold that is essential to allow this post-mitotic neuron migration, but also the communication between the different cell types. Coherent with this, it has been shown that **connexin 43** (Cx43) and **26** (Cx26) connect migrating neurons and RG fibers via gap junction dynamic adhesive contacts (Elias et al., 2007). Importantly, when either *Cx43* or *Cx26* is downregulated via shRNAs injected in mouse embryonic cortex, the neuron’s ability to migrate is reduced, without however, affecting the RG scaffold and expression of other cell-cell adhesion proteins. Also, as explained in section “Role of Secreted Proteins Derived From the CSF in the Formation and Maintenance of the RG Scaffold” (see BLBP section), neuron attachments to RG fibers are important for RG process elongation. In this context, it is important to mention that N-cadherin also plays a role in the attachment of the migrating neuron to the RG fiber, with its knockdown diminishing this interaction. The correct level of N-cadherin at the neuronal cell surface is mediated via endocytic pathways dependent on Rab GTPases (Kawauchi et al., 2010; Shikanai et al., 2011).

**Cdc42** is a small GTPase localized at the leading edge of basal radial fibers, where it allows the recruitment of protein complexes such as Par6-aPKC (Etienne-Manneville and Hall, 2003; Heasman and Ridley, 2008). Cdc42 plays a role in RG−RG interactions via inter-radial fibers, during the dynamic extension of the basal process (Figure 5). Indeed, *Cdc42* KO in the mouse leads to shorter basal processes which do not reach the pial surface during cortical development. The number of contacts between RG fibers is also reduced. Very little is known about the role of the inter-radial fiber on the scaffold, but there is a correlation between less inter-radial fibers and shorter RG basal processes (Yokota et al., 2010). Cdc42 signaling is known to be regulated via GSK3β phosphorylation, but the phenotype of the RG scaffold when GSK3 is pharmacologically inhibited is not the same as the *Cdc42* cKO phenotype. Indeed, although basal processes are shorter as in the *Cdc42* cKO, they are not well oriented at the pial surface after inhibition of GSK3 and the whole scaffold shows a wavy morphology. This suggests distinct roles for these two proteins influencing RG scaffolding and basal processes (Yokota et al., 2010).

As mentioned previously, in the Wnt signaling pathway **APC** has a role in the maintenance and extension of the RG scaffold. APC is localized in RG tips and in the soma. When it is specifically deleted in RGs *in vivo*, the scaffold is mis-oriented with basal processes not directed to the pial surface (Yokota et al., 2009). Over corticogenesis, the processes appear also shorter, suggesting a role for APC in maintenance of scaffold polarity but also fiber extension. APC is involved in the response of basal process extension via neuregulin 1 signaling (see section “Role of Secreted Proteins Derived From the CSF in the Formation and Maintenance of the RG Scaffold”), but also in the stability of microtubules at cell contacts (apical AJs and in basal RG endfeet at BM sites, Yokota et al., 2009). Indeed, APC is known to interact with microtubule proteins such as EB1 and microtubules themselves, and to allow the correct localization of polarity proteins (Numb, Cdc42) in subcellular compartments. Without APC, the integrity of apical and basal cell-cell contacts may hence be altered. This may explain why in its absence, basal fibers do not respond to neuregulin 1 since the interactions are not made correctly. However, its intrinsic role in microtubule stability may also play a role in this mechanism (Yokota et al., 2009).

## Further Factors Identified via Human Pathology

As previously mentioned, depletion of long range and short range factors can disrupt RG proliferation leading to microcephaly. Disruption of RG architecture on which neuronal migration relies, can also lead to other cortical malformations mentioned here, including human lissencephaly, polymicrogyria, and heterotopia. We describe the key features and genes involved in these disorders, shedding further light on external influences of neuronal migration.

### Apically Disrupted RGs

When RG architecture is perturbed apically it can lead to heterotopias associated with epilepsy and sometimes intellectual disability (Bizzotto and Francis, 2015). Firstly, perturbation of RGs and neuron migration can lead to **periventricular nodular heterotopia** (PH) where clusters of neurons are identified close to the ventricles. In PH models, during development abnormal clusters of progenitors and neurons are found trapped at the ventricular surface (Bizzotto and Francis, 2015; Table 2). Secondly, although **subcortical band heterotopia** (abnormal neuron clusters found within the white matter) is usually associated with an intrinsic problem in migrating neurons, other subcortical heterotopias (SH) can arise due to perturbed and apically detached RG, which subsequently perturb migration (Kielar et al., 2014; Stouffer et al., 2016).

**TABLE 2:** Genes mutated in human pathology associated with apical defects.

Gene	Pathology	OMIM_ number/acronym	OMIM neurological	LOF or GOF Model	Brain phenotype	Gene function	References
ARFGEF2/Arfgef2	Peri ventricular heterotopia	608097 PERI VENTRICULAR HETEROTOPIA WITH MICROCEPHALY; ARPHM; AUTOSOMAL RECESSIVE	Microcephaly, progressive Delayed psychomotor development Mental retardation, severe Seizures Hypsarrhythmia Quadriparesis Periventricular nodular heterotopia seen on MRI Thin corpus callosum	LOF Mouse models: gene-trap; early postnatal intraventricular injections of 40 μm brefeldin-A (BFA).	Gene-trap: Early embryonic lethality. BFA: heterotopic nodules below the ventricular surface; discontinuous N-cadherin staining	ADP-ribosylation factor guanine nucleotide-exchange factor-2; brefeldin A (BFA)-inhibited GEF2 protein (BIG2), which is required for vesicle and membrane trafficking from the *trans*-Golgi network (TGN)	Sheen et al., 2004; Grzmil et al., 2010
CTNNA2/Ctnna2	Pachygyria; Cerebellar hypoplasia	618174 CORTICAL DYSPLASIA, COMPLEX, WITH OTHER BRAIN MALFORMATION 9; CDCBM9, AUTOSOMAL RECESSIVE	Microcephaly, acquired Global developmental delay, Intellectual disability, severe Absent speech Inability to walk Ataxia Spastic tetraplegia Hyperreflexia Seizures, myoclonic, atonic, intractable Abnormal EEG Pachygyria Thickened cortex Thin CC Absent anterior commissure	LOF Cerebellar-deficient folia’ (cdf) mice	Cerebellar ataxia and hypoplasia. Cerebellar and hippocampal lamination defects	Alpha-N-catenin, cadherin-associated protein related; cytoskeleton protein anchoring cadherins	Cook et al., 1997; Schaffer et al., 2018
DCHS1/Dchs1	Periventricular heterotopia, van Maldergem	601390 VAN MALDERGEM SYNDROME 1; VMLDS1; AUTOSOMAL RECESSIVE	Mental retardation Intellectual disability Periventricular nodular heterotopia Subcortical band heterotopia Pachygyria Simplified gyral pattern Thin corpus callosum	LOF Dchs1-null embryonic mice; mouse IUE ShRNA; human *in vitro* organoid model	Early lethality; IUE: cells accumulated in the proliferative zones of the developing cortex. Changed proliferation, differentiation balance. Human: changed morphology of progenitor cells, defective migration of a subset of neurons, PH	Transmembrane cell adhesion molecule that belongs to the protocadherin superfamily. Apically located adhesive complex.	Cappello et al., 2013; Klaus et al., 2019
ECE2/Ece2	Periventricular heterotopia	None	None	LOF Mouse IUE and human cerebral organoid models	Ectopic localization of neural progenitors and neurons (including non-cell autonomous). Rosettes of progenitors and neurons in cortex. Perturbed ventricular surface, progenitor detachment.	Endothelin-converting enzyme 2; type II metalloprotease; Links cytoskeleton and adhesion. Regulates secretion of extracellular matrix molecules	Buchsbaum et al., 2020
EML1/Eml1	MEG, heterotopia	600348 BAND HETEROTOPIA; BH; AUTOSOMAL RECESSIVE	Macrocephaly Hydrocephalus Delayed development Intellectual disability Spasticity Seizures Sleep problems Subcortical band heterotopia Polymicrogyria Agenesis CC Dilated ventricles Behavioral problems	LOF *HeCo* heterotopic cortiex mice	Subcortical heterotopia; abnormal primary cilia	Microtubule-associated protein playing a role in trafficking from the Golgi apparatus.	Kielar et al., 2014; Uzquiano et al., 2019
ERMARD/Ermard/C6orf70	Periventricular heterotopia	615544 PERIVENTRICULAR NODULAR HETEROTOPIA 6; PVNH6; AUTOSOMAL DOMINANT	Delayed psychomotor development Seizures Delayed speech Hypsarrhythmia Hypoplastic corpus callosum, hippocampus and cerebellum Periventricular nodular heterotopia Polymicrogyria	LOF (haplo insuffi-ciency) IUE rat brain	Massive neuronal migration defect, significant arrest of cells within the ventricular zone, and development of heterotopic nodules along the walls of the lateral ventricles	Endoplasmic reticulum membrane-associated RNA degradation protein	Conti et al., 2013
FAT4/Fat4	Periventricular heterotopia, van Maldergem	615546 VAN MALDERGEM SYNDROME 2; VMLDS2; AUTOSOMAL RECESSIVE	Mental retardation Intellectual disability Periventricular nodular heterotopia Subcortical band heterotopia Thin corpus callosum	LOF Fat4-null mouse mutants; IUE mouse. Human *in vitro* organoid model.	Mouse mutants early lethality. IUE: cells accumulated in the proliferative zones of the developing cortex, heterotopia. Human organoid: disorganized germinal layer, premature delamination of progentors, abnormal neuronal migration, nodules	Member of a large family of protocadherins; role in vertebrate planar cell polarity	Cappello et al., 2013; Klaus et al., 2019
FLNA/FlnA	Periventricular heterotopia	300049 PERI VENTRICULAR NODULAR HETEROTOPIA 1; PVNH1; X-LINKED	Seizures, refractory to treatment Imaging shows non-calcified subependymal periventricular heterotopic nodules Mental retardation, mild Strokes due to coagulopathy Neuronal migration disorder	LOF FlnA knockout mice. Conditional mice (neural progenitors)	Knockout mice die at E14.5. Conditional mice have disrupted ventricular surface, perturbed intermediate progenitors. Exuberant angiogenesis.	Actin-binding protein making a link with plasma membrane proteins	Fox et al., 1998; Feng et al., 2006; Houlihan et al., 2016
GNAI2/Gnai2	Periventricular heterotopia	No obvious OMIM number Periventricular Nodular Heterotopia and Intellectual Disability, *de novo*	Intellectual disability Periventricular nodular heterotopia	LOF IUE knockdown mice	Delayed radial migration of excitatory neurons during corticogenesis, perhaps because of impaired morphology. No effects on proliferation or position of progenitors.	Guanine nucleotide binding protein, alpha inhibiting activity polypeptide 2. G-proteins transduce signals from seven−transmembrane− type receptors (G−protein−coupled receptors) to various downstream effectors	Hamada et al., 2017
GPSM2/Gpsm2/LGN	Periventricular heterotopia, PMG, Chudley-McCollough	604213 CHUDLEY-MCCULLOUGH SYNDROME; CMCS; AUTOSOMAL RECESSIVE	Hydrocephalus Ventricomegaly Intellectual disability rare Seizures rare CC abnormality Cerebellar hypo or dysplasia Obstruction of the foramen of Monro (variable) Subcortical nodular heterotopia Polymicrogyria Arachnoid cysts	LOF Drosophila mutant. Mouse knockout mutant.	Drosophila: mutant neuroblasts rapidly fail to self-renew. Randomized orientation of normally planar neuroepithelial divisions. Abnormally localized progenitors.	G-protein signaling modulator 2, Leu-Gly Asn repeat enriched protein. Modulates activation of G proteins which transduce extracellular signals received by cell surface receptors into integrated cellular responses. Involved in orientation of divisions	Lee et al., 2006; Konno et al., 2008; Doherty et al., 2012
HNRNPK/Hnrnpk	Au-Kline syndrome, Periventricular heterotopia	616580 AU-KLINE SYNDROME; AUKS; KABUKI-LIKE SYNDROME, AUTOSOMAL DOMINANT	Delayed psychomotor development Intellectual disability Poor speech High pain tolerance Nodular heterotopia (in 1 patient)	LOF (haplo insuffi-ciency) Mouse mutant	Down- regulation of hnRNPK in cultured hippocampal neurons by RNAi results in an enlarged dendritic tree and a significant increase in filopodia formation. Link to actin cytoskeleton.	Heterogeneous nuclear ribonucleoprotein K. Involved in chromatin remodeling, transcription, and mRNA splicing, translation, and stability. Pre-mRNA metabolism of transcripts containing cytidine-rich sequences.	Proepper et al., 2011; Lange et al., 2016
INTS8/Ints8	Periventricular heterotopia	618572 NEURODEVELOPMENTAL DISORDER WITH CEREBELLAR HYPOPLASIA AND SPASTICITY; NEDCHS; AUTOSOMAL RECESSIVE	Microcephaly, borderline Dysmorphic facial features Optic atrophy Hypertelorism Developmental delay Intellectual disability severe Inability to walk, talk Spastic paraplegia Seizures Cerebellar hypoplasia Pontine hypoplasia Brainstem hypoplasia Periventricular nodular heterotopia	LOF Drosophila mutant	Ectopic type II neuroblasts. Normally prevents de-differentiation of intermediate neural progenitors back into neural stem cells. IntS8 genetically interacts with ERM to suppress the formation of ectopic neuroblasts.	Integrator complex subunit. Associates with the C-terminal domain of RNA polymerase II large subunit. Mediates 3-prime end processing of small nuclear RNAs U1	Oegema et al., 2017; Zhang et al., 2019
KAT6B/Kat6b	Periventricular heterotopia	606170; 603736 GENITOPATELLAR SYNDROME; GTPTS; OHDO SYNDROME, SBBYS VARIANT; SBBYSS; AUTOSOMAL DOMINANT	Microcephaly Agenesis of corpus callosum Psychomotor retardation, severe Hypotonia Colpocephaly Periventricular neuronal heterotopia	LOF? Mouse gene-trap mutant. *Querkopf* mutant.	Homozygous die before weaning. Brain developmental defects. Less cells in cortical plate especially layer 5. Fewer interneurons.	Histone (lysine) acetyltransferase. Activated by the chromatin regulator Brpf1	Thomas et al., 2000; Clayton-Smith et al., 2011
MED12/med12	Heterotopia	305450; 309520 OPITZ-KAVEGGIA SYNDROME; OKS; LUJAN-FRYNS SYNDROME; X-LINKED	Macrocephaly Developmental delay Intellectual disability Neonatal hypotonia Seizures Hydrocephalus Agenesis CC Heterotopia Attention deficit disorder Hyperactivity Friendly, sociable personality (some) Aggressive behavior (some) Autistic-like behavior (some) Poor	LOF Zebrafish mutant and over-expression of *med12* RNA.	Embryos showed defects in brain, neural crest, and kidney development and do not survive beyond 1 week after fertilization. Re-expression of *med*12 RNA leads to premature neuronal differentiation.	Mediator of RNA polymerase II transcription. Coactivator of Sox9. Regulates the expression of distinct neuronal determination genes.	Hong et al., 2005; Wang et al., 2006; Caro-Llopis et al., 2016
		social interactions Emotional instability (some) Obsessive compulsive disorder (some) Poor impulse control (some) Low frustration tolerance (some) Psychosis (some)					
NEDD4L/Nedd4l	Periventricular heterotopia, PMG	617201 PERIVENTRICULAR NODULAR HETEROTOPIA 7; PVNH7; AUTOSOMAL DOMINANT	Delayed psychomotor development Intellectual disability Poor or absent speech Delayed or absent walking Seizures (in some patients) Periventricular nodular heterotopia Cortical dysplasia (in some patients) Thin corpus callosum (in some patients)	GOF IUE mouse to express mutant proteins. Knockdown.	Mutants: increased mitotic index, and arrest of neuronal cells within the ventricular and periventricular zone, depletion of neurons in the cortical plate. Terminal translocation disrupted? Knockdown - no differences.	E3 ubiqutin ligase. One target is the epithelial sodium channel (ENaC). Influences different signaling pathways. Player in regulation of the crosstalk between PI3K–mTORC2 and TGF-β–activin–Smad2–Smad3 (Smad2/3) signaling pathways	Broix et al., 2016
RPGRIP1L/Rpgrip1l/FTM/Ftm	Subcortical heterotopia	None	None	LOF Mouse IUE	Ectopic localization of neural progenitors Rosettes of progenitors in cortex. Perturbed ventricular surface, progenitor detachment.	Can associate with base of the primary cilia; Involved in proteasome degradation and autophagy	Uzquiano et al., 2019
TMTC3/Tmtc3/Smile	Periventricular heterotopia, cobblestone brain malformation;	617255 LISSENCEPHALY 8; LIS8; AUTOSOMAL RECESSIVE; PERIVENTRICULAR NODULAR HETEROTOPIA	Microcephaly Delayed psychomotor development Intellectual disability Poor or absent speech Seizures Appendicular spasticity Lissencephaly, cobblestone Polymicrogyria Ventricomegaly Abnormal myelination Nocturnal seizures Hypoplasia CC Hypo and dysplasia of the brainstem Hypo and dysplasia of the cerebellum Occipital encephalocele Autistic features	LOF *Smile* mouse mutant; Crispr/Cas9 *in vitro*. Fly model; post-mitotic neuron-specific knockdown	Mouse, early neonatal death; Fly, seizures, presynaptic function?	Transmembrane and tetratricopeptide repeat containing 3 gene. Positive regulator of the endoplasmic reticulum (ER) stress response. Also co-localization of TMTC3 in the rat brain with vesicular GABA transporter at pre-synaptic terminals. CDH and PCDH O-Man glycosylation.	Farhan et al., 2017; Larsen et al., 2017

*LOF, loss of function; GOF, gain of function; CC, corpus callosum; IUE, *in utero* electroporation; ShRNA, short hairpin RNA; PH, periventricular heterotopia. Human clinical information obtained from https://omim.org/.*

A number of PH genes highlighted here code for proteins regulating apical RG functions, with often as well evidence for a role in migrating neurons. PH is classically associated with mutations in *Filamin A*, coding for an actin cross-linking protein interacting with cell adhesion molecules such as integrins as well as other membrane proteins, enabling their anchoring to the cytoskeleton (Fox et al., 1998; Lian and Sheen, 2015; Table 2). Other PH proteins have been implicated in vesicle trafficking, e.g., ARFGEF2 required for trafficking from the Golgi apparatus (Sheen et al., 2004); and ERMARD and TMTC3, endoplasmic reticulum (ER) proteins (Conti et al., 2013; Larsen et al., 2017). *ARFGEF2* mutations can perturb proliferation and have been shown to affect the localization of cadherins and β catenin at the cell surface (Sheen et al., 2004), thus disrupting AJs. Mutations in α *N-catenin* (*CTNNA2*) also give rise to severe brain malformations (complex cortical dysplasia, Schaffer et al., 2018). The apical protocadherin receptor-ligand pair *DCHS1* and *FAT4* also show mutations in Van Maldergem syndrome which includes PH (Cappello et al., 2013). Acute knockdown in the mouse of these genes showed accumulation of cells in the VZ, as well as migration defects. Klaus et al. (2019) went on to show defective RG morphologies and transcriptional signatures, a discontinuous apical surface and slowed migration in human *in vitro* organoid models (Klaus et al., 2019). RG delamination most probably due to perturbed apical adhesion or signaling was also observed in these and other models (e.g., also with *EML*1 mutations giving rise to SH, Kielar et al., 2014) and this can lead to subtle or severe disruption of the ventricular surface, sometimes resulting in heterotopia.

Several PH proteins (e.g., GNAI2, GSPM2 involved in G-protein signaling) are likely to be involved in the transduction of extracellular signals to intracellular effectors (Lee et al., 2006; Hamada et al., 2017). Further proteins impact intracellular signaling (e.g., NEDD4L, Broix et al., 2016). Extracellular signaling was also revealed as important for RG function in the case of *ECE2* mutations (Buchsbaum et al., 2020). This enzyme localizes to the plasma membrane and its mutation in the developing mouse cortex led to RG delamination and the formation of rosettes (progenitors clustered in a circle within the tissue). Surrounding non-mutant cells also appeared to be affected (non-cell autonomous phenomenon). The ventricular surface showed morphological alterations suggesting a weakening of cell junctions and indeed proteomic analyses revealed deregulated ECM molecules, as well as cytoskeletal and cell adhesion proteins. Thus, transduction of extracellular signals as well as cell adhesion regulation are clearly important in RGs and migrating neurons, their disruption leading to PH (Lian and Sheen, 2015; Buchsbaum et al., 2020).

### Breakages in the BM and Cajal-Retzius Cells

**Cobblestone lissencephaly** is associated with disorganized cerebral and cerebellar cortices and multiple coarse gyri, with agyric regions (Table 3, OMIM; Guerrini and Dobyns, 2014). It is often included in a broader spectrum of disorders including muscular dystrophy and eye defects, as well as sometimes agenesis of the corpus callosum, cerebellar hypoplasia and hydrocephalus (OMIM; Guerrini and Dobyns, 2014). The dysfunctional mechanisms involve an over-migration of neurons at the pial surface, due to breaks in the cortical BM (Nickolls and Bonnemann, 2018). This has been associated with RG basal process end-feet that are not well attached to the ECM (e.g., laminin, Figure 3), leading to subsequent disintegration of the RG scaffold. In mouse models, mis-localization of Cajal-Retzius cells is also observed, in some models correlated with rostro-caudal and medio-lateral gradients of the lesions and the severity of the brain phenotype (Booler et al., 2016).

**TABLE 3:** Genes mutated in human pathology associated with basal defects.

Gene	Pathology	OMIM_ number/acronym	OMIM (neurological)	LOF or GOF Model	Brain phenotype	Gene function	References
ADGRG1 (GPR56), Gpr56	Polymicrogyria	606854; 615752 POLYMICROGYRIABI LATERAL FRONTOPARIE TALBFPP; POLYMICROGYRIABI LATERAL PERISYLVIAN; BPPR; AUTOSOMAL RECESSIVE	Developmental delay Psychomotor delay Intellectual disability, moderate to severe Seizures Cerebellar signs Pyramidal signs Polymicrogyria, most severe in the frontoparietal regions Polymicrogyria, anterior to posterior gradient Areas of dysmyelination on MRI Brainstem hypoplasia Cerebellar hypoplasia	LOF Mouse knockout	Neuronal ectopia in the cerebral cortex, a cobblestone-like cortical malformation.	7 transmembrane domains, as well as a mucin-like domain. Autoproteolytic cleavage to produce N-terminal adhesion ectodomain and transmembrane domain, which associate on cell surface. Receptor for collagens	Piao et al., 2004; Li et al., 2008; Paavola et al., 2011
ATP6V0A2/Atp6V0A2	Cobblestone brain malformation	219200 CUTIS LAXA, AUTOSOMAL RECESSIVE, TYPE IIA; ARCL2A	Microcephaly Delayed motor development Intellectual disability Seizures Hypotonia Partial pachygyria Cobblestone lissencephaly, posterior frontal and parietal regions Board and poorly defined gyri Polymicrogyria Dandy-Walker malformation	LOF Studies in the mouse (e.g., monoclonal antibody, anti-a2V)	Spontaneous abortions due to placental expression; role also in sperm	Integral membrane subunit of a vacuolar-type proton pump (H (+)-ATPase or V-ATPase) for acidification of diverse organelles and vesicles. Involved in N-glycosylation at the level of processing in the Golgi apparatus	Kornak et al., 2008; Van Maldergem et al., 2008; Jaiswal et al., 2015
B3GALNT2/B3Galnt2	Cobblestone brain malformation	615181 MUSCULAR DYSTROPHY-DYSTROGLYCANOPATHY (CONGENITAL WITH BRAIN AND EYE ANOMALIES), TYPE A, 11; MDDGA11; AUTOSOMAL RECESSIVE	Delayed psychomotor development, severe Lack of acquisition of motor milestones Severe cognitive impairment Hydrocephalus Polymicrogyria Cobblestone lissencephaly Frontotemporal leukoencephalopathy Cerebellar dysplasia Pontocerebellar hypoplasia Cerebellar cysts	LOF Zebrafish knockdown	Retinal degeneration, hydrocephalusand severely impaired motility.	Transmembrane protein, beta-1,3-N-Acetyl Galactosaminyltransferase 2; Adds galactose residues, to synthesize poly-N-acetyllactosamine.	Stevens et al., 2013
B4GAT1/B3GNT1/B4Gat1/B3Gnt1	Cobblestone brain malformation	615287 MUSCULAR DYSTROPHY-DYSTROGLYCANOPATHY (CONGENITAL WITH BRAIN AND EYE ANOMALIES), TYPE A, 13; MDDGA13; AUTOSOMAL RECESSIVE	Lack of psychomotor development Hydrocephalus Anencephaly Occipital encephalocele Enlarged ventricles Seizures Spasticity Agenesis of the corpus callosum Brainstem hypoplasia Cerebellar hypoplasia Cortical dysplasia Cobblestone lissencephaly Nodular heterotopia Dandy-Walker malformation	LOF Mouse ENU, null mutation (B3gnt1 LacZ/LacZ) and hypomorphs B3gnt1LacZ/M155T	Null: E9.5 lethality. Hypomorphs: Defective glycosylation of alpha-dystroglycan. Congenital muscular dystrophy. Radial glial endfoot detachment and cobblestone-like phenotype.	Transmembrane protein i-beta-1,3-N-acetylglucosaminyl transferase. N-acetylglucosamine residues added to synthesize poly-N-acetyllactosamine, a linear carbohydrate that can be incorporated into either N- or O-linked glycans.	Wright et al., 2012; Buysse et al., 2013; Shaheen et al., 2013
COL3A1/Col3A1	Cobblestone brain malformation	618343 POLYMICROGYRIA WITH OR WITHOUT VASCULAR-TYPE EHLERS-DANLOS SYNDROME; (PMGEDSV); AUTOSOMAL RECESSIVE	Delayed motor development Impaired intellectual development Seizures Speech delay Polymicrogyria Cobblestone-like malformation of the cortex Anterior to posterior gradient Enlarged ventricles Cerebellar	LOF Mouse knockout	Early postnatal death. At E18.5, cobblestone like cortical malformation with pial breakdown in the basement membrane, neuronal overmigration, RG detachment,	ECM molecule present in basement membranes	Liu et al., 1997; Jeong et al., 2012; Horn et al., 2017; Vandervore et al., 2017
			hypoplasia Cerebellar cysts Brainstem hypoplasia Abnormal corpus callosum White matter abnormalities		and formation of marginal zone heterotopias		
COL4A1/Col4A1	Cobblestone-like brain malformation	POLYMICROGYRIA SCHIZENCEPHALYPOREN CEPHALY, WWS or MEB, AUTOSOMAL RECESSIVE	Variable	LOF Mouse mutant Col4a1 + /Δex40 (splice acceptor mutation)	Homozygous lethal. Heterozygote mice develop porencephaly secondary to focal disruptions of vascular basement membranes, Col4a1 + /Δex40 mice also show pial basement membrane disruptions and cerebral cortical lamination defects	ECM molecule, ubiquitously present in basement membranes. Interacts with COL4A2	Gould et al., 2005; Labelle-Dumais et al., 2011; Cavallin et al., 2018; Zagaglia et al., 2018
DAG1/Dag1	Cobblestone brain malformation	616538 MUSCULAR DYSTROPHY-DYSTROGLYCANOPATHY (CONGENITAL WITH BRAIN AND EYE ANOMALIES), TYPE A, 9; MDDGA9; AUTOSOMAL RECESSIVE	Macrocephaly Delayed psychomotor development, severe Lack of speech Poor head control Hydrocephalus Thin cortical layer Polymicrogyria Frontal agyria Migration defects Dilated ventricles Thin corpus callosum Kinking of the pons and brainstem Hypoplastic cerebellar vermis Cerebellar cysts White matter abnormalities Leukodystrophy Cystic lesions Intracranial calcifications	LOF Mouse KO and cKO; KI	KO, embryonic lethal. Brain-specific deletion: discontinuous *glia limitans*, perturbed cortical layering, fusion of cerebral hemispheres and cerebellar folia, aberrant migration of granule cells. KI: muscular dystrophy and neurologic motor impairment, glycosylation by LARGE is decreased.	Glycoprotein, membrane associated. Interacts with ECM molecules	Williamson et al., 1997; Henry and Campbell, 1998; Moore et al., 2002; Hara et al., 2011; Geis et al., 2013
FKRP/Fkrp	Cobblestone brain malformation (WWS, MEB or less severe form)	613153 MUSCULAR DYSTROPHY-DYSTROGLYCANOPATHY (CONGENITAL WITH BRAIN AND EYE ANOMALIES), TYPE A, 5; MDDGA5; AUTOSOMAL RECESSIVE	Intellectual disability, profound Delayed motor development, severe Hydrocephalus Cobblestone lissencephaly Agyria Cerebellar cyst Absence of the cerebellar vermis Pontine hypoplasia Cerebellar hypoplasia Cerebellar dysplasia Pachygyria Hypoplastic brainstem Ventricular dilatation Absence of the corpus callosum White matter abnormalities Dandy-Walker malformation Hyporeflexia	LOF Mouse hypomorphic knockin. Zebrafish morpholino	KI mice die around birth. decreased muscle mass, perturbation of the limiting membrane of the eye, and a disturbance in neuronal migration. Zebrafish: muscle and eye phenotype	Golgi-resident glycosyltransferase. Could impact dystroglycan maturation.	Brockington et al., 2001; Ackroyd et al., 2009; Kawahara et al., 2010
FKTN/Fktn	Cobblestone brain malformation (Fukuyama congenital muscular dystrophy, WWS, MEB or less severe form)	253800 MUSCULAR DYSTROPHY-DYSTROGLYCANOPATHY (CONGENITAL WITH BRAIN AND EYE ANOMALIES), TYPE A, 4; MDDGA4; AUTOSOMAL RECESSIVE	Intellectual disability Poor motor development Polymicrogyria Leptomeningeal thickening Focal interhemispheric fusion Low density white matter Cobblestone lissencephaly Pachygyria Agyria Agenesis of the corpus callosum Encephalocele (rare) Hydrocephalus Cerebellar cysts Seizures Hyperekplexia (rare) Pyramidal tract hypoplasia Brainstem hypoplasia Cerebellar hypoplasia Hypo- or areflexia	LOF Mouse	Laminar disorganization of the cortical structures in the brain with impaired laminin assembly, focal interhemispheric fusion, and hippocampal and cerebellar dysgenesis. Loss of laminar structure in the retina,	Golgi-resident glycosyltransferase. Secreted protein. Expressed in Cajal Retzius cells and cortical neurons. In ECM, modifies glycosylation of DAG1	Kobayashi et al., 1998; Sasaki et al., 2000; Hayashi et al., 2001; Beltran-Valero De Bernabe et al., 2002; Takeda et al., 2003
ISPD/Ispd	Cobblestone brain malformation	614643 MUSCULAR DYSTROPHY-DYSTROGLYCANOPATHY (CONGENITAL WITH BRAIN AND EYE ANOMALIES), TYPE A, 7; MDDGA7; AUTOSOMAL RECESSIVE	Macrocephaly Intellectual disability, profound Hydrocephalus Ventriculomegaly Encephalocele Dandy-Walker malformation Cobblestone lissencephaly Agyria Pachygyria Polymicrogyria Hypoplasia of the corpus callosum Partial agenesis of the corpus callosum Cortical thinning Subcortical heterotopia Cerebellar hypoplasia Brainstem hypoplasia Brain vascular anomalies (rare) Areflexia	LOF Zebrafish morpholino-based knockdown; Mouse ENU mutant (stop mutation)	Zebrafish: Hydrocephalus and incomplete brain folding, with significantly reduced eye size. Mouse: lethal P1, defective axon guidance. Cobblestone-like phenotype. Reduced glycosylation of dystroglycan	ISPD has an isoprenoid synthase domain characteristic of nucleotide diP-sugar transferases	Roscioli et al., 2012; Vuillaumier-Barrot et al., 2012; Wright et al., 2012
LAMA2/Lama2	Polymicrogyria, Cobblestone brain malformation (some patients)	607855 MUSCULAR DYSTROPHY, CONGENITAL MEROSIN-DEFICIENT, 1A; MDC1A; AUTOSOMAL RECESSIVE	White matter hypodensities seen on MRI Abnormal cortical gyration (rare) Seizures (rare) Intellectual disability (rare) Lissencephaly (rare)	LOF Mouse KO or transgenic	Lethlality. Symptoms of congenital muscular dystrophy. Full or partial laminin deficiency. Brain phenotype may require other gene mutations	ECM molecule, alpha-2 laminin subunit	Guo et al., 2003; Dominov et al., 2005; Nelson et al., 2015; Oliveira et al., 2018
LAMB1/Lamb1	Cobblestone brain malformation; no eye and muscle phenotypes.	615191 LISSENCEPHALY 5; LIS5; AUTOSOMAL RECESSIVE	Macrocephaly due to hydrocephalus Psychomotor retardation Intellectual deficiency progressive Hypotonia Seizures Spastic paraplegia Cobblestone lissencephaly (posterior brain regions more affected than anterior regions) Subcortical band heterotopia Occipital encephalocele Cerebellar hypoplasia Brainstem hypoplasia Leukoencephalopathy White matter cysts Porencephaly White matter atrophy, progressive	LOF? Mouse - spontaneous mutant (stop codon) leads to modest truncation	Homozygous lethal. Dystonia-like phenotype in heterozygote state.	ECM molecule, beta-1 laminin subunit, regulates axon guidance	Radmanesh et al., 2013; Liu et al., 2015; Tonduti et al., 2015
LAMC3/Lamc3	Polymicrogy riaoccipital pachygyria	614115 CORTICAL MALFORMATIONS, OCCIPITAL; OCCM; AUTOSOMAL RECESSIVE	Seizures, absence Seizures, tonic-clonic (1 patient) Delayed psychomotor development (1 patient) Autonomic symptoms Pachygyria, occipital Polymicrogyria, occipital EEG abnormalities	LOF Mouse	Retinal phenotype. Gene expressed in vessels and meninges	ECM molecule, gamma-3 laminin subunit	Denes et al., 2007; Barak et al., 2011; Radner et al., 2013; Zambonin et al., 2018
LARGE/Large	Cobblestone brain malformation (WWS, MEB or milder)	613154 MUSCULAR DYSTROPHY-DYSTROGLYCANOPATHY (CONGENITAL WITH BRAIN AND EYE ANOMALIES), TYPE A, 6; MDDGA6; AUTOSOMAL RECESSIVE	Intellectual disability Areflexia Cobblestone lissencephaly Ventricular dilatation Absence of the cerebellar vermis Hypoplasia and dysplasia of the cerebellum Hydrocephalus White matter changes Pontine hypoplasia Dandy-Walker malformation (rare)	LOF Mouse myd mutation deletion in Large gene	myd mice have abnormal neuronal migration in the cerebral cortex, cerebellum, and hippocampus, and show disruption of the basal lamina.	Transmembrane protein N-acetylglucosaminyl transferase. Adds a glycan repeat to dystroglycan	Michele et al., 2002; Longman et al., 2003; Van Reeuwijk et al., 2007
POMGNT1/Pomgnt1	Cobblestone brain malformation (WWS, MEB or milder)	253280 MUSCULAR DYSTROPHY-DYSTROGLYCANOPATHY (CONGENITAL WITH BRAIN AND EYE ANOMALIES), TYPE A, 3; MDDGA3; AUTOSOMAL RECESSIVE	Microcephaly Intellectual disability, severe Hypotonia, severe Seizures Hydrocephalus Lack of motor development (WWS) Disorganized brain cytoarchitecture Ventricular dilatation White matter changes Cerebellar hypoplasia Cerebellar dysplasia Brainstem hypoplasia Brainstem concavity Flattening of the pons Complete or partial absence of the corpus callosum Cobblestone lissencephaly, type II Pachygyria Polymicrogyria Cerebellar cysts	LOF Mouse knockout (gene-trap)	Abnormal cortex, disappearance of molecular layer I (overmigration); cerebral hemispheres fused. Hippocampal dysplasia and scalloped DG. Enlarged lateral ventricles	Type II transmembrane protein. O-mannose beta-1,2-N-acetyl glucosaminyl transferase, participates in O-mannosyl glycan synthesis	Yoshida et al., 2001; Liu et al., 2006; Bouchet et al., 2007
POMGNT2/Pomgnt2, GTCD2/Gtcd2 (AGO61)	Cobblestone brain malformation (WWS)	614830 MUSCULAR DYSTROPHY-DYSTROGLYCANOPATHY (CONGENITAL WITH BRAIN AND EYE ANOMALIES), TYPE A, 8; MDDGA8; AUTOSOMAL RECESSIVE	Lack of psychomotor development Hydrocephalus Enlarged ventricles Cobblestone lissencephaly Cerebellar hypoplasia	LOF Zebrafish morpholino-based knockdown; Mouse knockout	Zebrafish: hydrocephalus, ocular defects, and muscular dystrophy. Mouse: lethal P1, abnormal basal lamina formation and a neuronal migration defect. RG endfoot detachment.	Endoplasmic reticulum (ER)-resident protein with N terminal signal peptide, that catalyzes the second step of the O-mannosyl glycosylation in the mucin-like domain of alpha-dystroglycan. Glycosyltransferase-like domain-containing protein-2, O-mannose β-1,4-N-acetyl glucosaminyltransferase.	Manzini et al., 2012; Yagi et al., 2013
POMK/Pomk, SGK196	Cobblestone brain malformation	615249 MUSCULAR DYSTROPHY-DYSTROGLYCANOPATHY (CONGENITAL WITH BRAIN AND EYE ANOMALIES), TYPE A, 12; MDDGA12; AUTOSOMAL RECESSIVE	Microcephaly, progressive (1 patient) Delayed psychomotor development, severe Psychomotor retardation, severe Loss of ambulation Poor speech Seizures (1 patient) Hydrocephalus Cerebellar hypoplasia Brainstem hypoplasia (1 patient) Cobblestone lissencephaly (1 patient) Agenesis of the corpus callosum (1 patient) Agyria (1 patient) Brain hypomyelination	LOF Zebrafish morpholine knockdown, Mouse knockout	Zebrafish: small head, delayed ocular development, shortened thicker tail; Mouse, neuronal migration defects, cerebellar dysplasia, hydrocephlaus	Protein-O-mannose kinase. Transmembrane protein with extracellular kinase-like domain, phosphorylates the 6-position of O-mannose.	Vogel et al., 2012; Jae et al., 2013; Yoshida-Moriguchi et al., 2013; Di Costanzo et al., 2014; Von Renesse et al., 2014
POMT1/Pomt1	Cobblestone brain malformation (WWS, MEB and a less severe form)	236670 MUSCULAR DYSTROPHY-DYSTROGLYCANOPATHY (CONGENITAL WITH BRAIN AND EYE ANOMALIES), TYPE A, 1; MDDGA1; AUTOSOMAL RECESSIVE	Microcephaly Hypotonia, severe Seizures Hydrocephalus Ventricular dilatation White matter changes Cerebellar hypoplasia Cerebellar dysplasia Brainstem hypoplasia Flattening of the pons Agenesis of the CC Occipital encephalocele Meningoencephalocele Thin cortical mantle Cobblestone lissencephaly Agyria Pachygyria Fused hemispheres Posterior fossa cysts Virtual absence of pyramidal tracts Polymicrogyria (MEB) Cerebellar cysts (MEB)	LOF Mouse	Embryonic lethal.	Integral membrane protein. O-mannosyl transferase that catalyzes the first step in the synthesis of the O-mannosyl glycan found on alpha-dystroglycan (see also POMT2)	Beltran-Valero De Bernabe et al., 2002; Willer et al., 2004; Bouchet et al., 2007
POMT2/Pomt2	Cobblestone brain malformation (WWS, MEB and a less severe form)	613150 MUSCULAR DYSTROPHY-DYSTROGLYCANOPATHY (CONGENITAL WITH BRAIN AND EYE ANOMALIES), TYPE A, 2; MDDGA2; AUTOSOMAL RECESSIVE	Microcephaly Intellectual disability, Hypotonia, severe Hydrocephalus Ventricular dilatation Cerebellar hypoplasia Cerebellar dysplasia Brainstem hypoplasia Flattening of the pons Cobblestone lissencephaly, type II Smooth, thin mantle Aplasia of the CC Encephalocele (1 patient, MEB) Cerebellar cysts (MEB) Pachygyria with frontoparietal involvement (MEB) Polymicrogyria (MEB) Periventricular white matter changes (MEB) Diffuse white matter changes (MEB) Seizures (MEB)	LOF Mouse knockout (constitutive and conditional)	KO embryonic lethal. cKO Emx1-Cre: neocortical dysplasia (over-migration), migration failure in cerebellum, hippocampal dysplasia, displaced Cajal–Retzius cells, disruption of the BM. Hypo glycosylation of alpha-DG.	Integral membrane protein. Sequence similarity with a family of protein O-mannosyl transferases, that catalyze the first step in the synthesis of the O-mannosyl glycan found on alpha-dystroglycan	Willer et al., 2002; Van Reeuwijk et al., 2005; Bouchet et al., 2007; Hu et al., 2011
RELN/reln	Lissencephaly, Pachygyria, cerebellar hypoplasia	257320 LISSENCEPHALY SYNDROME, NORMAN-ROBERTS TYPE NORMAN-ROBERTS SYNDROME; AUTOSOMAL RECESSIVE	Microcephaly Lissencephaly, type I Thick cerebral cortex Cerebellar hypoplasia	LOF Mouse spontaneous “reeler”	Impaired motor coordination, tremors, and ataxia. Neurons fail to reach their correct locations in the developing brain, disrupting the organization of the cerebellar and cerebral cortices and other laminated regions.	Secreted glycoprotein expressed in Cajal-Retzius cells	D’Arcangelo et al., 1995; Hong et al., 2000
SRD5A3/Srd5A3	Cobblestone brain malformation	612379 CONGENITAL DISORDER OF GLYCOSYLATION, TYPE Iq; CDG1Q; AUTOSOMAL RECESSIVE	Brachycephaly Intellectual disability Hypotonia Delayed motor development Pituitary gland hypoplasia Polymicrogyria, frontal Cerebellar vermis hypoplasia	LOF Mouse knockout	Embryonic lethal at day E12.5; upregulation of genes involved in regulation of the unfolded protein response (ER - related to role of N-glycan?)	Polyprenol reductase. Reduction of polyprenol is the major pathway for dolichol biosynthesis during N-glycosylation	Cantagrel et al., 2010
TBC1D20/Tbc1D20	Cobblestone brain malformation	615663 WARBURG MICRO SYNDROME 4; WARBM4; AUTOSOMAL RECESSIVE	Postnatal microcephaly Brachycephaly Congenital hypotonia, axial or generalized Postnatal development of hypertonic extremities Spastic quadriplegia Speech severely limited Seizures Cortical atrophy Hypoplastic CC Bilateral frontoparietal polymicrogyria Widened lateral ventricles Progressive cerebellar atrophy Mega cisterna magna Autistic features	LOF Mouse spontaneous “*blind-sterile”*	Nuclear cataracts and male infertility; no obvious brain abnormalities	TBC1 domain family member; TBC (Tre2, Bub2, and Cdc16) domains found in most Rab-GTPase-activating proteins (GAPs), which are important in vesicular membrane transport. Associates with the ER.	Sklan et al., 2007; Liegel et al., 2013
TMTC3/Tmtc3/Smile	Cobblestone brain malformation; no eye and muscle phenotypes.	617255 LISSENCEPHALY 8; LIS8; AUTOSOMAL RECESSIVE; PERIVENTRICULAR NODULAR HETEROTOPIA	Microcephaly (in some patients) Delayed psychomotor development Delayed walking Intellectual disability Poor or absent speech Seizures Appendicular spasticity Lissencephaly, cobblestone Polymicrogyria Ventriculomegaly Abnormal myelination (in some patients) Hypoplasia of the CC Hypoplasia of the brainstem Hypoplasia of the cerebellum Dysplasia of the brainstem Dysplasia of the cerebellum Occipital encephalocele (in some patients) Autistic features (in some patients)	LOF *Smile* mouse mutant; Crispr/Cas9 *in vitro*. Fly model; post-mitotic neuron-specific knockdown	Mouse, early neonatal death; Fly, presynaptic function?	Transmembrane and tetratricopeptide repeat containing 3 gene. Positive regulator of the endoplasmic reticulum (ER) stress response. Also co-localization of TMTC3 in the rat brain with vesicular GABA transporter at pre-synaptic terminals. CDH and PCDH O-Man glycosylation.	Racape et al., 2011; Yun and Vu, 2012; Jerber et al., 2016; Farhan et al., 2017; Larsen et al., 2017
TMEM5/Tmem5	Cobblestone brain malformation	615041 MUSCULAR DYSTROPHY-DYSTROGLYCANOPATHY (CONGENITAL WITH BRAIN AND EYE ANOMALIES), TYPE A, 10; MDDGA10; AUTOSOMAL RECESSIVE	Cobblestone lissencephaly Occipital neural tube defects Cerebellar dysplasia Macrocephaly (in some patients)	LOF None		Transmembrane protein believed to have glycosyltransferase function	Vuillaumier-Barrot et al., 2012; Jae et al., 2013
VLDLR/Vldlr	Lissencephaly; abnormal neuron migration	224050 CEREBELLAR ATAXIA, MENTAL RETARDATION, AND DYSEQUILIBRIUM SYNDROME 1; CAMRQ1; AUTOSOMAL RECESSIVE	Psychomotor retardation Mental retardation Poor speech development Gait ataxia Truncal ataxia Disturbed equilibrium Quadrupedal gait (in some) Intention tremor Dysarthria Dysmetria Dysdiadochokinesis Hypotonia Hyperreflexia Broad-based gait Seizures (rare) Cortical gyral simplification Pachygyria Cerebellar hypoplasia Cerebellar ataxia Small brainstem	LOF Vldlr knockout mouse (or double knockout with ApoeR2)	Invasion of migrating neurons in the MZ. Double knockout with ApoeR2 leads to inverted and disorganized cortical layers	Receptor for reelin expressed on migrating neurons	Trommsdorff et al., 1999; Hack et al., 2007; Valence et al., 2016

*WWS, Walker-Warburg syndrome; MEB, Muscle eye brain; KO, knockout; cKO, conditional knockout; KI, knockin; LOF, loss of function; GOF, gain of function; ECM, extracellular matrix; BM, basement membrane; CC, corpus callosum. Human clinical information obtained from https://omim.org/.*

As mentioned previously, receptors and glycoproteins present on RG basal membranes normally make interactions with the ECM, helping to form and maintain the BM (Nickolls and Bonnemann, 2018). Mutations in some of these molecules (see below) leads to a cobblestone lissencephaly phenotype (Table 3). In other cases, a **polymicrogyria** phenotype in human patients is associated with a cobblestone-like phenotype revealed in mouse models. Indeed, polymicrogyria, associated with multiple small folds at the surface of the brain (Francis et al., 2006) also often involves over-migration and BM breakages. In this disorder, regions of fused gyri within the brain parenchyma can also contain BM components (Squier and Jansen, 2014). Thus, cobblestone lissencephaly is associated with polymicrogyria and indeed, often the two are found together (Squier and Jansen, 2014). These disorders can also often be associated with other neuronal migration defects such as periventricular or subcortical heterotopia (Table 3). We examine here causes of these linked disorders, from human genetics and animal model data.

Central to cobblestone lissencephaly hypotheses is post-translational regulation of dystroglycan (coded by DAG1, dystrophin-associated glycoprotein 1), a glycoprotein present in RG basal processes, that acts as an anchor point with the ECM (Booler et al., 2016), being also important for BM structure (Henry and Campbell, 1998). It normally gets cleaved giving rise to the peripheral membrane protein α-DG and the transmembrane protein β-DG. α-DG undergoes O-linked mannosylation allowing its binding to ECM proteins such as laminin (which has α, β and γ subunits, Burgeson et al., 1994), agrin, neurexin, pikachurin, and perlecan. The ER is involved in trafficking and glycosylating secretory pathway cargo. β-DG interacts with the actin cytoskeleton via dystrophin, utrophin and plectin, and thus α-DG and β-DG link the cytoskeleton to the ECM (Booler et al., 2016).

Muscle eye brain disease (MEB), Fukuyama congenital muscular dystrophy (FCMD), and Walker Warburg syndrome (WWS) have each been associated with aberrant glycosylation of α-DG (e.g., Kobayashi et al., 1998; Yoshida et al., 2001; Michele et al., 2002). Abnormal dystroglycan−ligand interactions underlie the pathogenic mechanism of muscular dystrophy as well as brain abnormalities. Rare mutations in *DAG1* (coding for dystroglycan, Hara et al., 2011; Geis et al., 2013), laminin subunit genes (e.g., *LAMA2*, *LAMB1*, and *LAMC3*, Barak et al., 2011; Radmanesh et al., 2013; Nelson et al., 2015; Zambonin et al., 2018) and genes coding for proteins likely to influence glycosylation and maturation of α-DG as well as potentially other proteins (ATP6V0A2, B4GAT1, B3GALNT2, FKTN, FKRP, ISPD, LARGE, POMK, POMT1, POMT2, POMGNT1, POMGNT2, SRD5A3, TMEM5, Kobayashi et al., 1998; Brockington et al., 2001; Yoshida et al., 2001; Beltran-Valero De Bernabe et al., 2002; Willer et al., 2004; Van Reeuwijk et al., 2005, 2007; Cantagrel et al., 2010; Manzini et al., 2012; Roscioli et al., 2012; Vuillaumier-Barrot et al., 2012; Buysse et al., 2013; Jae et al., 2013; Stevens et al., 2013 and see Table 3) can give rise cobblestone lissencephaly and/or polymicrogyria.

Several other genes involved in cobblestone phenotypes (*TBC1D10, TMTC3*, Liegel et al., 2013; Jerber et al., 2016) code for proteins which are likely to be involved in membrane trafficking and/or the ER stress response. Interestingly, TMTC3 has also been associated with 0-mannosyl glycosylation in the ER of the cell adhesion cadherins and proto-cadherins, but not α-DG (Larsen et al., 2017). As mentioned previously, cadherins are involved in RG apical cell-cell contacts, which may hence be disrupted if glycosylation does not occur correctly. Several patients with PH, already associated with abnormal RG contacts and neuronal migration (Cappello et al., 2013), exhibit mutations in *TMTC3* (Larsen et al., 2017). Thus, most likely apical (periventricular) as well as basal interactions require correct glycosylation.

*GPR56* mutations were identified in bilateral frontoparietal or perisylvian polymicrogyria (Piao et al., 2004; Bae et al., 2014). GPR56 is a member of an adhesion family of G protein-coupled receptors (GPRs), expressed on the basal membrane of RGs. Mouse mutants show a cobblestone-like phenotype, with abnormal basal RG processes, a disrupted BM, with neuronal over-migration and Cajal Retzius cell disruption (Li et al., 2008). Type III collagen, as well as being important in connective tissue including blood vessels, is one of the major constituents of the pial BM and a ligand for GPR56 (Luo et al., 2011). Although several disorders are associated with altered levels of the collagen COL3A1 (Kuivaniemi and Tromp, 2019), some mutations give rise to frontal predominant bilateral polymicrogyria or a cobblestone−like cortical malformation (Horn et al., 2017; Vandervore et al., 2017). COL3A1 is the α1 chain of type III collagen, an ECM protein. COL4A1, the α1 chain of type IV collagen, is also a crucial component of the BM. Cobblestone lissencephaly, polymicrogyria, schizencephaly or porencephaly were observed in certain patients with *COL4A1* mutations (Labelle-Dumais et al., 2011; Cavallin et al., 2018; Zagaglia et al., 2018). Cavallin et al. (2018) discuss prenatal stroke and suggest that hydranencephaly, schizencephaly, porencephaly and polymicrogyria represent a continuum of brain injury depending on the timing and the severity of the insult. Both genetic as well as environmental factors may hence contribute to these disorders. Interestingly, *COL4A2* mutations were also identified in a patient presenting heterotopia in addition to bilateral polymicrogyria suggesting perturbed neuronal migration (Cavallin et al., 2018).

By activating signaling cascades, Reelin acts at the terminal steps of neuronal migration, notably terminal translocation, without however, influencing RG-guided migration (Sekine et al., 2014). As mentioned previously however (section “Role of Secreted Proteins Derived From the CSF in the Formation and Maintenance of the RG Scaffold”), Reelin may also influence RG basal processes at least in some species. Lower concentrations of Reelin are also present in the lower IZ, in this case influencing the neuronal multipolar-bipolar transition (Sekine et al., 2014). Mutations have been identified in *Reelin* in autosomal recessive pachygyria associated with cerebellar hypoplasia (Hong et al., 2000). *Reeler* mutant mice show a similar combination of defects, including disorganized and inverted cortical lamination (D’Arcangelo et al., 1995). There are also patients which exhibit mutations in the Reelin receptor gene, *very low density lipoprotein receptor*, *VLDLR* (Valence et al., 2016). Mutations give rise to the CARMQ1 syndrome (cerebellar ataxia and mental retardation with or without quadrupedal locomotion), which can include mild simplification or thickening of cortical gyri (Valence et al., 2016). *Vldlr* mouse mutants show an invasion of neurons in the MZ (Hack et al., 2007) and double KO with a second Reelin receptor gene, *ApoER2*, phenocopies the cortical disorganization observed in *reeler* mutants (Trommsdorff et al., 1999). Thus, extracellular Reelin via the above-mentioned receptors, is critical for neuron migration and notably translocation in the most superficial regions of the developing cortex, as well as potentially maintenance of the RG scaffold (Weiss et al., 2003).

## Conclusion and Perspectives

Overall, in this review we summarize the variety of different factors which together regulate the formation and the maintenance of RG scaffolding. The need for this variety, ranging from secreted factors in the eCSF to very local cell to cell contacts, is probably due to the unique morphology of RGs. Indeed, they spread from one side of the developing cortex to the other, and are therefore exposed to very different environments. RGs have basal endfeet in contact with secreted factors derived from the meninges and from cells migrating from remote places, such as Cajal Retzius cells or neurons, and at the same time aRGs have their cell bodies and primary cilia in contact with other RG and the eCSF (Figure 1). For the stability of the whole cortical architecture, which is based on RGs, it therefore seems to be essential that there are multiple spatial environments which signal to the different RG compartments. aRGs will also differ from bRGs in this respect and it is also clear that environments change over time further regulating these essential cell types.

It appears that there is not a single mechanism that governs the RG scaffold but instead elaborated mechanisms involving numerous actors. However, although this review is not exhaustive, it is striking that unlike what it is already known for neuronal growth and guidance, there is little research which has up till now identified specific modulators of RG growth and maintenance. We attempted here to identify and group such factors. RGs have to grow a long process to reach a remote location, crossing a relatively thick tissue, however, they are often seen as a constant scaffold acting as a support for neuronal migration. The RG scaffold is not though as static as we might imagine. Indeed, evidence provided by the work of Yokota et al. (2010), showed using videomicroscopy that RG processes, endfeet and radial fiber interactions are constantly dynamic. The function of this dynamic behavior could impact neuronal migration at the local level to finely regulate neuron positioning in the CP and hence the structure of the brain. How and why the RG scaffold is plastic still remains to be investigated. Experiments taking into account this dynamic behavior could help acquire further information to distinguish factors involved in RG growth aspects from those involved in maintenance.

Radial glia are not only defined by their morphology. They are also able to adapt their gene expression to match the changing cues in their environment and to adjust their cellular responses (Telley et al., 2019). The timing of these mechanisms is crucial as cues can be produced in waves during development (Chau et al., 2015). How these genic modulations occur still needs to be studied. However, factors influencing chromatin and RNA processing are likely to play a role (e.g., it is notable that there are several PH genes involved in these processes, including HNRNPK, INTS8, KAT6B, MED12, Clayton-Smith et al., 2011; Caro-Llopis et al., 2016; Lange et al., 2016; Oegema et al., 2017, see Table 2), although for the moment in relatively unknown ways. Further studies across corticogenesis could shed further light on such mechanisms. Transcriptomic or proteomic experiments related to these genes could further identify key targets (acting on adhesion and/or signaling) impacting progenitor and/or neuron migration function at different timepoints.

When we think of the role and formation of the RG scaffold, another aspect to consider is the evolution from lissencephalic species to gyrencephalic species, especially with respect to the appearance of bRGs. Indeed, related to this relatively new progenitor type, whose somata lie in more basal areas of the developing cortex of gyrencephalic species, the RG network is not the same as in lissencephalic species. Some questions are: what are the relationships between aRG basal processes and bRG basal processes? Do they interact? Do they respond in the same manner to extracellular cues? How is bRG maintenance initially put in place, since these cells do not receive the same extracellular cues as their apical counterparts? Are there specific extracellular cues regulating bRGs, forming a special niche in the center of the developing cortex? For instance, a study identified the specific role of PDGFD signaling in the maintenance of bRG in human cortical development compared to mouse progenitors (Lui et al., 2014). Pollen et al. found several genes specific to bRGs which are highly related to ECM interactions. For instance tenascin C (TNC) is specific to bRGs and has the ability to bind to PTPRZ1, syndecans or integrins (Pollen et al., 2015). Moreover as mentioned earlier Notch is important for RG maintenance in mouse but recently a study showed that three paralogs of human-specific NOTCH2NL are essential to control the proliferation/differentiation balance of human RGs, which can be directly linked with the expansion of the human cortex (Fiddes et al., 2018). The function of the bRG scaffold is indeed still mysterious although it is widely assumed to contribute to gyrification during evolution. These ideas and relevant mechanisms are discussed in a review on the role of bRG morphology in cortical development and evolution (Kalebic and Huttner, 2020).

Radial glia are very unique and fascinating cells: the correct orchestration of their formation, organization, and localization is crucial for correct cortical development. Since the discovery of these cells, much work has been done to understand better how these progenitors communicate and interact with their environment but still a lot remains to be investigated. This review highlights a level of complexity inherent to these cells with respect to the impact of extracellular cues on their development and maintenance. Further understanding this aspect will help clarify mechanisms involved in health and neurodevelopmental disorders.

## Author Contributions

JF wrote the sections “Role of Secreted Proteins Derived From the CSF in the Formation and Maintenance of the RG Scaffold” and “Secreted Factors From Close Range Cells” and designed all the figures and parts of Table 1. DZ wrote the section “Further Factors Identified via Human Pathology” and generated Table 1. FF wrote the section “Role of Cell to Cell and Cell to ECM Contacts in the Formation and Maintenance of the RG Scaffold and Proliferation” and generated Tables 2, 3. All authors contributed to the article and approved the submitted version.

## Conflict of Interest

The authors declare that the research was conducted in the absence of any commercial or financial relationships that could be construed as a potential conflict of interest.
